# Dual Pathways, One Framework: Theoretical Insights into the Benzimidazole × Benzodiazepine Crossroads from *o*‑Phenylenediamine and 2‑Cyanoacrylate Derivatives

**DOI:** 10.1021/acsomega.5c05925

**Published:** 2025-10-21

**Authors:** Ramon S. da Silva, Ana J. F. Souza, Diego P. Sangi, Rodrigo G. Amorim

**Affiliations:** † Departamento de Física, Universidade Federal de Juiz de Fora, Juiz de Fora 36036-330, Minas Gerais, Brazil; ‡ Departamento de Química—Instituto de Ciências Exatas—ICEx, 28110 Universidade Federal Fluminense, Volta Redonda 27213-145, Rio de janeiro, Brazil; § Departamento de Física—Instituto de Ciências Exatas—ICEx, 28110 Universidade Federal Fluminense, Volta Redonda 27213-145, Rio de janeiro, Brazil

## Abstract

Ketene dithioacetals represent a significant class of molecules with a pivotal role in organic synthesis, particularly as versatile building blocks for the development of novel pharmaceutical compounds. We employed density functional theory (DFT) calculations in both ethanol and the gas phase to elucidate the specific mechanism of the reaction between methyl 2-cyano-3,3-bis­(methylthio)­acrylate and *o*-phenylenediamine. This approach provides a theoretical basis for understanding the system’s behavior and reactivity at the molecular scale. Our study evaluates the competition between two reported products: 4-methylsulfanyl-2-oxo-2,5-dihydro-1*H*-benzo­[*b*]­[1,4]­diazepine-3-carbonitrile and methyl 2-cyano-2-(1,3-dihydro-2*H*-benzo­[*d*]­imidazole-2-ylidene)­acetate. The present findings reveal that both reactions are exothermic, with the formation of methyl 2-cyano-2-(1,3-dihydro-2*H*-benzo­[*d*]­imidazole-2-ylidene)­acetate being more thermodynamically favorable. This combination of experimental evidence and computational analysis bridges the gap between synthetic applications and mechanistic understanding, enhancing the design and optimization of organic reactions.

## Graphical abstract

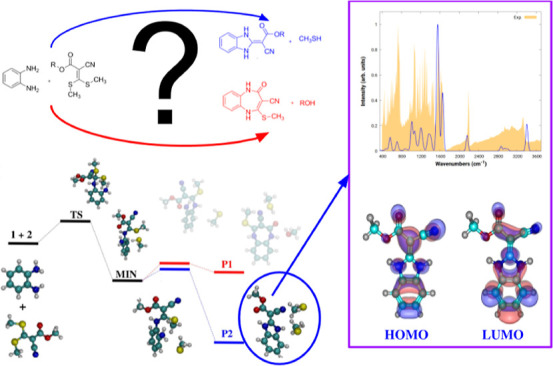

## Introduction

Heterocyclic compounds are extensively studied, due their great importance in pharmacy design, especially being used as scaffolds to drug discovery. Heterocyclic structures are considered privileged by their capacity to furnish ligands to different biological targets, attributed to their relative conformational stability and charge distribution, which contribute to enhancing pharmacodynamic properties.^1–4^

Benzodiazepines were the first heterocyclic compounds to be recognized as privileged structures by Evans et al.^5^ at 1988, having compounds with various biological activities as action on neuro system,^6,7^ as antifungal,^8^ bactericides,^9^ anticancer^10,11^ and antiviral agents.^12^ Benzimidazoles are another class of organic compounds considered privileged scaffolds to research for new drugs, because it has a lot of biological applications such as anticancer, antibacterial, and antifungal.^13–17^

Ketene dithioacetals have widespread use in organic chemistry, serving as important building blocks for obtaining heterocyclic scaffolds using various methods.^18,19^ The presence of electron withdrawing groups on the α carbon to thioacetal open the theoretical possibility to competitive transformations in reactions with 1,2 diamines as *o*-phenylenediamine.^20^ A double vinylic substitution mechanism on dithioacetal allows a [4 + 1] cyclization producing benzimidazole, while a mono vinylic substitution followed by amidation produces a [4 + 3] cyclization obtaining benzodiazepine.

In an paper published in 1992, Huang and Wang^21^ discuss the reaction between 1,2-diamines and ketene dithioacetals, and based on experimental observations concluded that benzimidazoles are obtained from *o*-phenylenediamine and ketene dithioacetals containing two electron withdrawing groups, while benzodiazepines are produced when ketene dithioacetal contain only one substituent.

Recently, Baliza et al.^22^ synthesized several heterocyclic compounds by performing double vinylic substitution in ketene dithioacetals and investigated their anticancer activity. In this study, methyl 2-cyano-2-(1,3-dihydro-2*H*-benzo­[*d*]­imidazole-2-ylidene)­acetate (**P2**) was synthesized from methyl 2-cyano-3,3-bis­(methylthio)­acrylate (**2**) and *o*-phenylenediamine (**1**), using microwave irradiation to promote the reaction in 1 h. Alternatively, the same reaction was also conducted using conventional heating over 4 h. To our surprise, in 2019, Misra and co-workers^23^ used similar experimental conditions to synthesize 4-methylsulfanyl-2-oxo-2,5-dihydro-1*H*-benzo­[*b*]­[1,4]­diazepine-3-carbonitrile (**P1**) (Figure 1).

**Figure 1:**
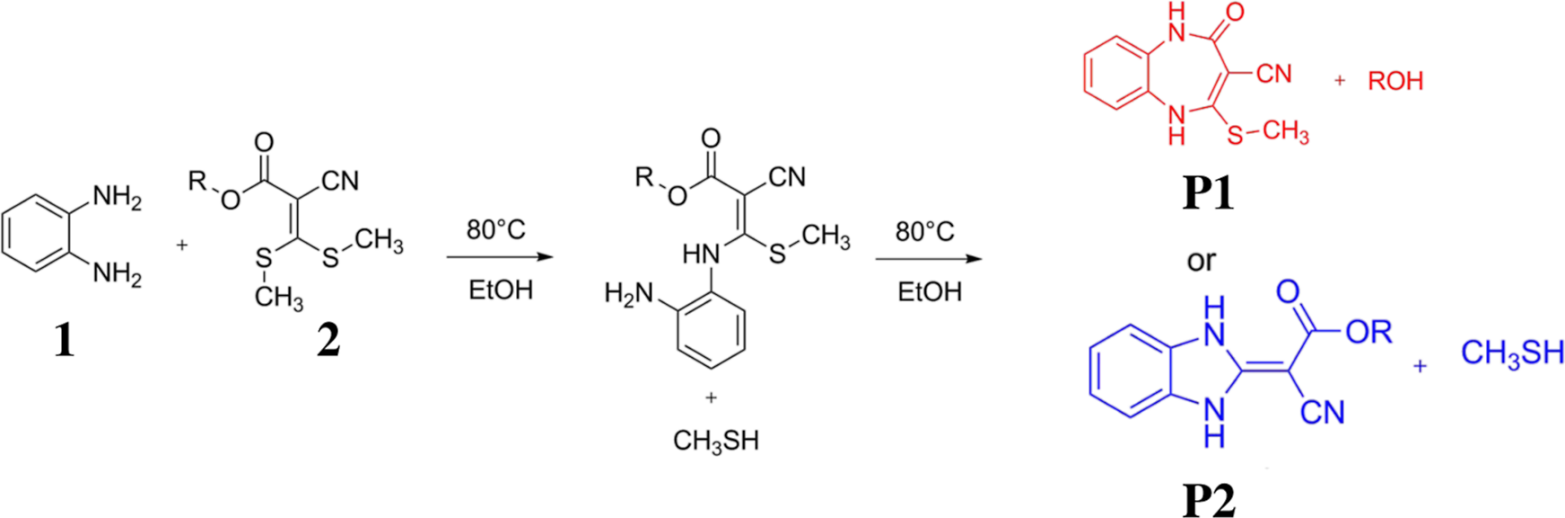
Reaction between 2-cyano-3,3-bis­(methylthio)­acrylate and *o*-phenylenediamine.

To gain deeper insight into the reaction between methyl 2-cyano-3,3-bis­(methylthio)­acrylate and *o*-phenylenediamine, we proposed a theoretical mechanism to explore the formation of methyl 2-cyano-2-(1,3-dihydro-2*H*-benzo­[*d*]­imidazole-2-ylidene)­acetate and 4-methylsulfanyl-2-oxo-2,5-dihydro-1*H*-benzo­[*b*]­[1,4]­diazepine-3-carbonitrile. To this end, we performed quantum chemistry calculations to estimate the relative energies of the intermediates and products, identifying which reaction pathway is thermodynamically favored.

## Computational Details

All quantum chemistry calculations were carried out using quantum chemistry suite.^24,25^ In this work, the global optimizer algorithm (GOAT),^26^ implemented in the ORCA 6.0 program, was employed to identify the lowest-energy conformers of reactants, products, and transition states using the GFN2-xTB methodology.^27^ GOAT calculations were performed to explore all conformers within a 6.0 kcal/mol energy window relative to the global minimum. The lowest-energy conformations were subsequently optimized using density functional theory (DFT) calculations.

Final single-point energy calculations were obtained using the hybrid Becke3-Lee–Yang–Parr (B3LYP)^28^ density functional (20% Hartree–Fock exchange) combined with Ahlrichs’ def2-TZVPP basis set,^29^ which includes high angular momentum polarization functions for all atoms. This basis set was contracted to 5s3p2d1f/_*C*/*N*_ and 3s2p1d_*H*_. For the sake of comparison, we also optimized the geometries using the correlation-consistent cc-pVTZ (VTZ) basis set,^30^ contracted to 4s3p2d1f/_*C*/*N*_ and 3s2p1d_*H*_. The calculations were accelerated using the RI-J approximation, with the def2/J auxiliary basis set^31^ employed for Ahlrichs’ basis sets and the cc-pVTZ/C auxiliary basis set for the Dunning cc-pVTZ basis set. All computations utilized the DefGrid2 numerical integration grid, and the SCF convergence criterion was set to VeryTight for all DFT calculations.

Noncovalent dispersion interactions are fundamental components of van der Waals forces that play a vital role in stabilizing molecular structures. In this work, these interactions were evaluated using the tom-pairwise dispersion corrections with Becke-Johnson damping, also referred to as D3BJ.^32,33^ The conductor-like polarizable continuum model (CPCM)^34^ was employed with ethanol (ϵ = 24.3) as the solvent, consistent with the experimental conditions, to enhance the realism of our calculations. We calculate the solvation free energy, Δ*E*_Solv_, defined as the difference between the free energy of the solvated system, obtained in the optimized geometry *R*_Solv_, and the free energy of the isolated system in vacuum, *E*_0_, determined in the coordinates *R*_0_.^35^

Numerical harmonic frequency calculations confirmed that the final optimized geometries correspond to local minima on the potential energy surface, as indicated by the presence of only real harmonic frequencies. In contrast, the transition state (TS1) was characterized by a single imaginary frequency, consistent with its identification as a first-order saddle point. Normal mode vibrational assignments were performed using the Chemcraft program.^36^

The DFT reactivity descriptors such as electronegativity (χ), chemical hardness (η), chemical potential (μ_0_), softness (*S*) and electrophilicity index (ω) were calculated using the energies of the HOMO and LUMO molecular orbitals. The energy difference between these orbitals defines the energy gap (*E*_gap_). The ionization potential (*I*) represents the minimum energy required to remove an electron from a neutral molecule, forming a cation. According to Koopmans’ theorem, this can be approximated by the highest occupied molecular orbital (HOMO) energy

1 $$I\approx -\epsilon_{HOMO}$$

Similarly, the electron affinity (*A*) corresponds to the energy change when a neutral molecule gains an electron to form an anion, which relates to the lowest unoccupied molecular orbital (LUMO) energy

2 $$A\approx -\epsilon_{LUMO}$$

The electron charge transfer capacity is best characterized by the electrophilicity index (ω)

3 $$\omega ={\mu_{0}}^{2}/2\eta$$

where μ_0_ is the chemical potential given by

4 $$\mu_{0}=-\chi$$

and

5 χ=(I+A)/2

The chemical hardness denoted by η is calculated according to equations

6 η=(I−A)/2

The softness was calculated using the relation

7 S=1/2η

while the maximum amount of electronic charge that an electrophile can accept is given by^37^

8 $$\Delta N_{\max}=-\left(\mu_{0}/\eta \right)$$

## Results and Discussion

### Synthesis of Methyl 2-Cyano-2-(1,3-dihydro-2*H*-benzo­[*d*]­imidazole-2-ylidene)­acetate

Following the procedure described by Baliza et al.,^22^ a mixture of *o*-phenylenediamine (**1**) (1 mmol) and methyl 2-cyano-3,3-bis­(methylsulfanyl)­acrylate (**2**) (1 mmol) (1 mmol) was refluxed in ethanol (5 mL) for 4 h. The solvent was removed by rotary evaporation, and the crude product was purified by a chromatographic column using dichloromethane as solvent. The purified products were then analyzed using ^1^H and ^13^C nuclear magnetic resonance, infrared spectroscopy and mass spectrometry. Methyl 2-cyano-2-(1,3-dihydro-2*H*-benzo­[*d*]­imidazole-2-ylidene)­acetate (**P2**): Yield 86%. Melting point: 278–280 °C (decomposition). ^1^H NMR (300 MHz, DMSO-*d*_6_): δ 12.34 (s, 2H); 7.40 (dd, *J* 5.5 and 3.2 Hz, 2H); 7.22–7.12 (m, 2H); 3.66 (s, 3H). ^13^C NMR (75 MHz, DMSO-*d*_6_): δ 167.66; 152.91; 131.37; 123.28; 119.75; 111.89; 51.47; 50.95. IR (ATR) (ν_max_/cm^–1^): 3325, 3105, 2195, 1622, 1568, 1068, 733. MS (*m*/*z*, (%)): 215(0); 157(100); 103(53); 63(52).

Ketene dithioacetals serve as conjugated substrates that undergo nucleophilic addition with amines. As illustrated in Figure 2 for the reaction between *o*-phenylenediamine (**1**) and 2-cyano-3,3-bis­(methylthio)­acrylate (**2**), the methylsulfanyl groups are substituted via an addition–elimination mechanism.

**Figure 2:**
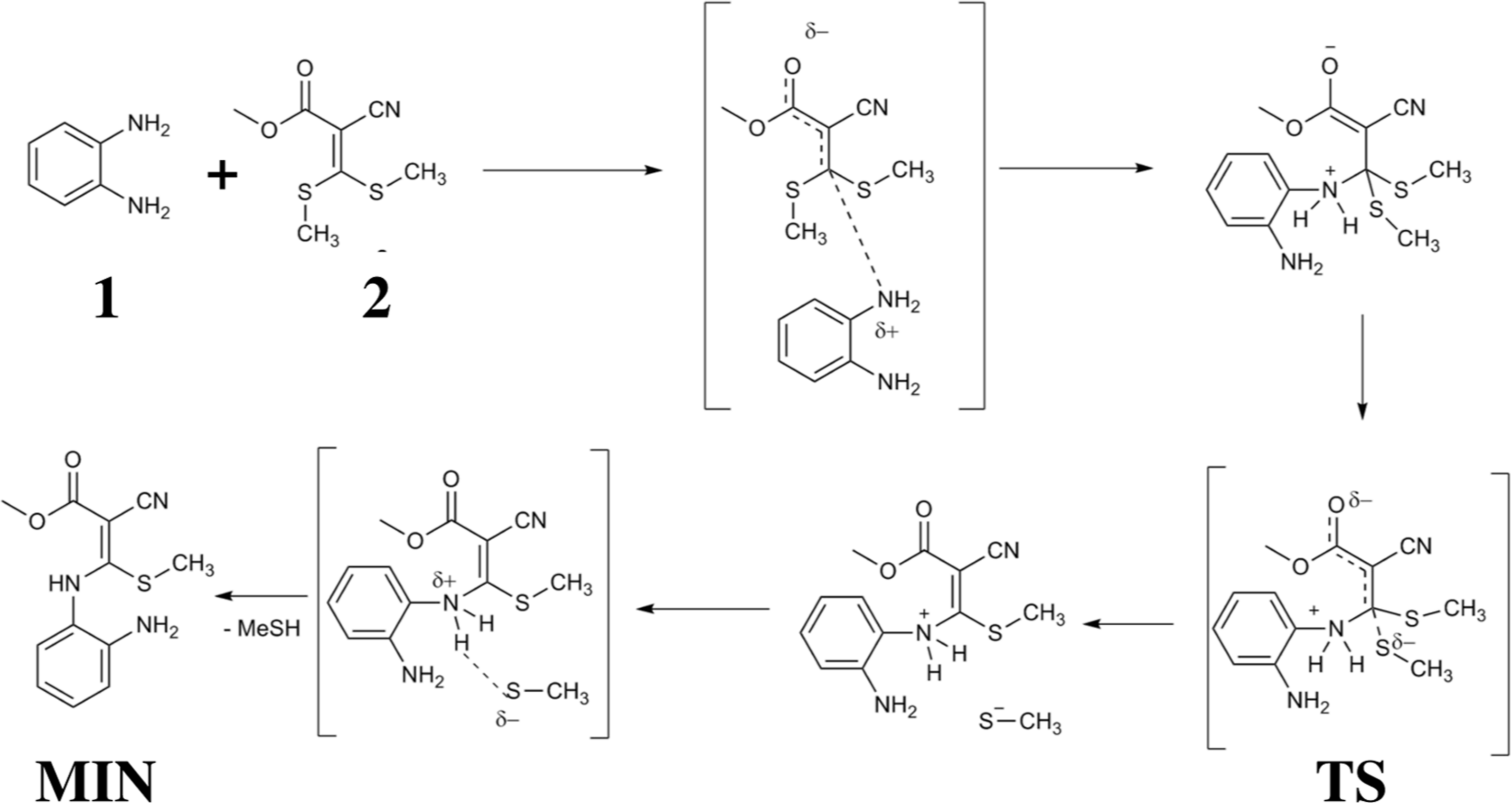
Reaction mechanism of *o*-phenylenediamine (**1**) and 2-cyano-3,3-bis­(methylthio)­acrylate (**2**).

Until the completion of the first vinylic substitution and formation of an adduct (**MIN**), the mechanism is the same to obtain benzodiazepine (**P1**) or benzimidazole (**P2**). After that, two reaction paths are possible: (1) second vinylic substitution and formation of compound 3 (Figure 3), (2) condensation with substituent ester group producing an amide and obtaining compound 4 (Figure 3).

**Figure 3:**
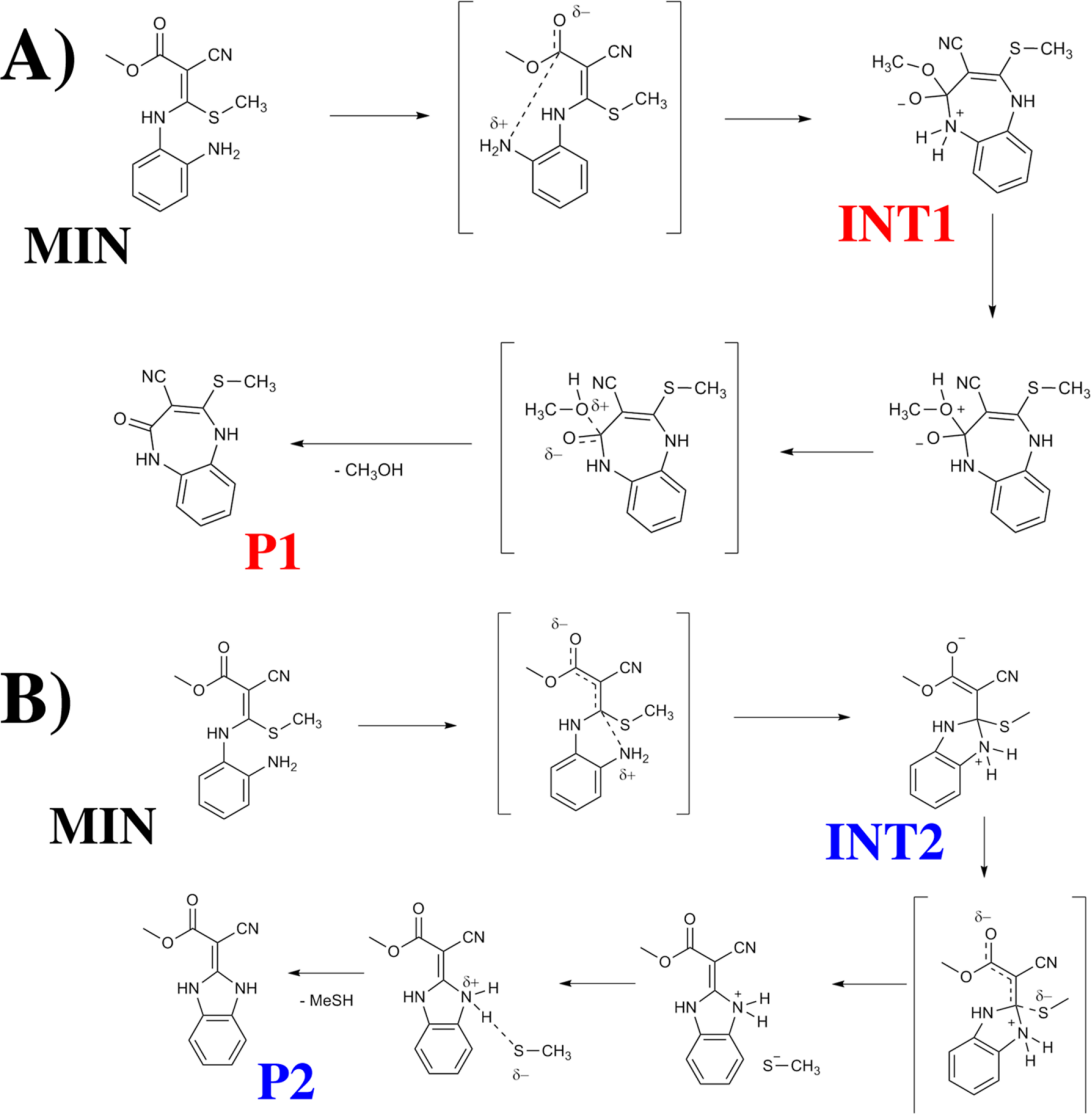
Reaction mechanism of 2-cyano-3,3-bis­(methylthio)­acrylate and *o*-phenylenediamine. (A) Formation of benzodiazepine **P1**, (B) formation of benzimidazole **P2**.

To determine the relative energies of reaction intermediates and products, we performed structural optimizations using density functional theory (DFT) calculations. The results are discussed in the following sections.

### Stationary Points

Figure 4 presents the optimized structures obtained at the B3LYP-D3BJ/def2-TZVPP level of theory, highlighting selected bond lengths and bond angles. It is important to emphasize that these geometries result from a more detailed analysis based on the structural schemes shown in Figures 2 and 3. Cartesian coordinates for all optimized structures are provided in the Supporting Information.

**Figure 4:**
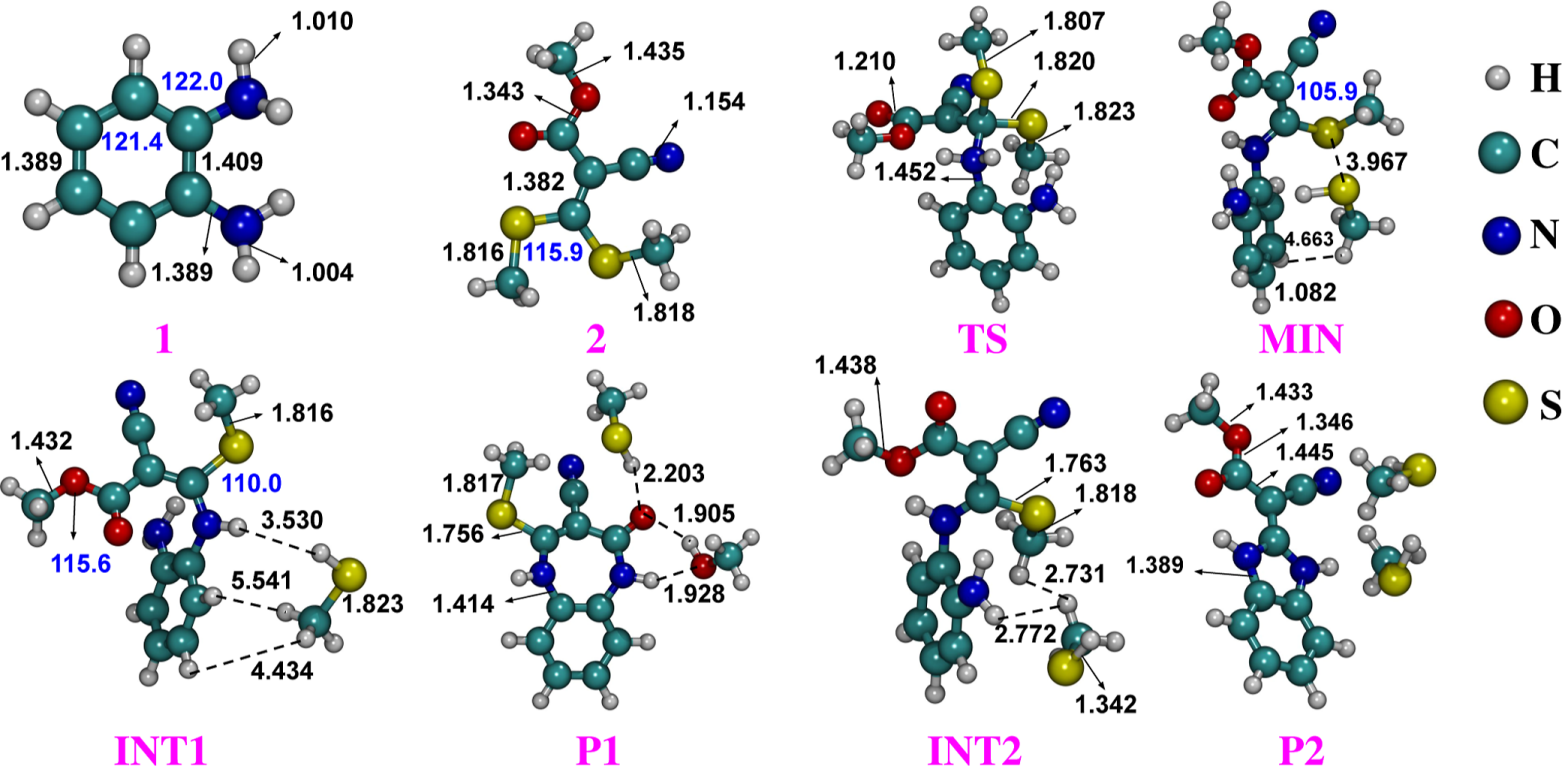
Optimized geometries using the B3LYP-D3BJ/def2-TZVPP level of theory. Selected bond distances are given in Å, and bond angles in degrees (in blue).

The optimized structure of *o*-phenylenediamine (compund **1**) is almost planar. Our GOAT calculations identified two conformers, whose structures are presented in Figure S1 of the Supporting Information. The conformers exhibit similar geometries, differing primarily in the orientation of the amino group (−NH_2_) at position 2. The higher-energy conformer lies just 2.0 kcal/mol above the global minimum.

The C–N and N–H bond distances in the minimum energy structure exhibit bond distances of 1.399 Å and 1.010 Å, respectively. The internal ring angle (C–C–C) is found to be 121.4°. The present data are in quantitative agreement with the DFT numbers reported by Ullah et al.^38^ Valiev and Minaev^39^ reported similar parameters (d­(C–C) = 1.4 Å and d­(C–H) = 1.084 Å) in their study of interactions between benzene and molecular oxygen using wave function-based methods. Second-order perturbation theory and coupled cluster methods yielded results consistent with the present data.^40^ The wave function and DFT methods have been employed by Varandas^41,42^ to investigate the electronic structure and geometry of C-bearing compounds. For melamine (C_3_N_6_H_6_), their coupled cluster CCSD­(T)/VDZ calculations provide d­(C–N) = 1.353 Å, d­(N–H) = 1.011 Å, and ∠H–N–H ≈ 115.3°,^41^ consistent with our findings.

Atomic charges were computed via Mulliken population analysis providing quantitative assessment of intramolecular charge distribution. The results are displayed in Figure S2 of the Supporting Information. Calculations performed with the def2-TZVPP basis set reveal that the nitrogen atoms carry the most negative charges (approximately – 0.34*e*), consistent with their role as electron-donating sites. Notably, the hydrogen atoms exhibit positive partial charges, confirming their role as electron acceptors in this system.

For methyl 2-cyano-3,3-bis­(methylthio)­acrylate, eight distinct conformations were identified through gas-phase GOAT calculations. For clarity, four representative structures are shown in Figure S3 of the Supporting Information. The molecule features a cyano (−C≡N) group and two methylthio (−S–CH_3_) substituents attached to an acrylate ester backbone. A visual inspection of this figure reveals a slight rotation of the methyl group connected to the oxygen atom from plot (A) to (B). The most significant structural differences are observed in plots (C) and (D), which exhibit energies of 3.6 and 3.9 kcal/mol, respectively, relative to the lowest-energy conformer.

The B3LYP-D3BJ/def2-TZVPP optimized geometry for compound **2** yields C­(β-carbon)–S bond distances of 1.743 Å. The C–S bond lengths computed for methylthio structures are ∼1.816 Å, while C–H bond lengths are close to 1.089 Å. These values are in good agreement with those recently reported for 1,1-bismethylsulfanyl-2-nitroethylene,^43^ a related sulfur-containing compound. The obtained C≡N bond length of 1.154 Å is shorter than the experimental value of 1.172 Å^44^ reported for free cyanide in its ground X^2^Σ^+^ electronic state. Both Methylthio moieties are bonded to the same carbon (β-carbon), forming an ∠S–C–S bond angle of 115.9°. This value differs by only 0.2° from that computed using the def2-TZVPP basis set with ethanol as the solvent. In general, the ∠H–C–H bond angles are in the range of 110°.

The net atomic charges of compound **2** were determined using Mulliken population analysis, with the results presented in Figure S4 of the Supporting Information. For clarity, the positions of α-carbon and β-carbon are explicitly labeled in the figure. It can be observed that the most negative charge is localized on the oxygen atom of the carbonyl (−C=O) within the ester functional group. All hydrogen atoms in the molecule display positive charges of approximately +0.12*e*, confirming their electron-deficient character and role as acceptor sites.

The solvation free energies of reactants **1** and **2** in ethanol were determined using the CPCM method. For *o*-phenylenediamine, our computations lead to Δ*E*_Solv_ = −9.5 kcal/mol. The Δ*E*_Solv_ value of −10.7 kcal/mol calculated for methyl 2-cyano-3,3-bis­(methylthio)­acrylate (**2**) is similar to that computed for solute **1**.

We now turn to the analysis of the transition state (TS). In this case, the GOAT calculations were restricted to conformers within a 3 kcal/mol window from the global minimum, resulting in the identification of 32 distinct structures. The TS one showed in Figure 4 was confirmed as a first-order saddle point on the potential energy surface by the presence of a single imaginary frequency in the vibrational spectrum. The optimized TS structure, obtained at the B3LYP-D3BJ/def2-TZVPP level of theory, exhibits an imaginary frequency of −73.8 cm^–1^. When solvent effects are considered, this value increases to −126.3 cm^–1^. Additionally, a vibrational frequency of −64.3 cm^–1^ was obtained using the cc-pVTZ basis set, further supporting the identification of the TS.

The atomic charges corresponding to the transition state are presented in Figure S5 of the Supporting Information. As shown, the nitrogen atom N(7) acquires a positive charge due to its involvement in four covalent bonds, resulting in a tetravalent N^+^ center. This observation aligns with the mechanism illustrated in Figure 2. The oxygen atom labeled O(33) bears the most negative charge, estimated at −0.33*e*, which further decreases to −0.45*e* when solvent effects are included. This negatively charged oxygen is likely to engage in electrostatic interactions with the hydrogen atoms bonded to N(7), suggesting the formation of hydrogen bonds.

Upon examination of Figure 4, it becomes clear that the N–H bond distances within the amino groups increase by ≈0.012 Å, while the C­(β-carbon)-NH_2_ are elongated by 1.452–1.389 = 0.063 Å compared to the reactant geometries. The optimized C=O and C≡N bond lengths calculated by def2-TZVPP basis set are 1.210 and 1.158 Å, respectively. These values increase by 0.014 and 0.003 Å, respectively, when solvent effects are incorporated.

Our DFT calculations identified a van der Waals (vdW) minimum geometry (**MIN**) comprising unbound methanethiol (SHCH_3_), in concordance with the reaction scheme proposed in Figure 2. As shown in Figure 4, the SHCH_3_ molecule is formed by the C–S bond cleavage in the TS structure, accompanied by the transfer of a hydrogen atom (or proton transfer) from N(7) (see Figure S5 of the Supporting Information) to the sulfur atom of the resulting methylthio moieties. The S–H, C–H, C–S bond distances in SHCH_3_ structure computed using the def2-TZVPP basis set are 1.344, 1.086, and 1.824 Å, respectively. These values agree well with the experimental data for the isolated methanethiol of d­(S–H) = 1.335 Å, d­(C–H) = 1.092 Å, and d­(C–S) = 1.814 Å.^45^ The N–C­(β-carbon) bond length was shortened from 1.452 Å in the transition state to 1.418 Å in the vdW minimum. The S···S intermolecular distance in this structure was estimated to be 3.967 [3.978] Å using the def2-TZVPP [VTZ] basis set. Upon inclusion of ethanol as a solvent, this distance increased to 4.104 Å. The calculated ∠C­(β-carbon)–S–C in the **MIN** structure is 105.4°, differing by only 2.0° from the corresponding angle in the TS geometry.

We additionally performed GOAT calculations to identify the most stable conformer among the remaining structures shown in schemes 2 and 3. For **P1** product, we identified 104 distinct conformers within a 3 kcal/mol energy window of the global minimum. Representative structures (four conformers) are displayed in Figure S6 of the Supporting Information. This Figure reveals a clear trend toward hydrogen bond O···H formation between the benzodiazepine and other molecular species in the system. For product **P2**, we found 98 conformers within a 3 kcal/mol energy window relative to the global minimum. Four representative structures are shown in Figure S7 of the Supporting Information, demonstrating varying spatial arrangements of the two methanethiol product groups.

In general, we found two different intermediates, denoted here as **INT1** e **INT2**. In both cases, no imaginary frequencies were found, confirming that they correspond to intermediates on the potential energy surface rather than transition states. Selected bond distances and bond angles of these structures are presented in Figure 4. A close inspection of Figure 4 reveals that the C–O bond distance in **INT1** structure (1.347 Å) is ∼0.01 Å longer than that corresponding bond in **MIN** (1.338 Å), suggesting possible bond weakening and imminent cleavage. This process is likely to lead to the formation of a methoxy group in product **P1**. Note also the presence of an open seven-membered ring structure containing carbon and nitrogen atoms. As discussed in the Introduction, a seven-membered ring with two nitrogen atoms is characteristic of benzodiazepine species. On the other hand, the C­(β-carbon)-S bond distance was estimated to be 1.749 Å in the vdW minimum. This value increases ≈0.015 Å in the stationary poitnt **INT2**, suggesting potential bond cleavage and the formation of a methylthio moieties (SCH_3_).

### Competing DFT Reaction Mechanisms: Benzimidazole × Benzodiazepine Formation

Table 1 presents the energy differences for all stationary points, referenced to the dissociation limit **1** + **2**, obtained from the sum of the reactants’ energies. The corresponding total and zero-point energies (ZPE) for each species are reported in Table S1 of the Supporting Information. Figure 5 displays the schematic potential energy diagram for the reaction between *o*-phenylenediamine (**1**) and methyl 2-cyano-3,3-bis­(methylthio)­acrylate (**2**) calculated using the B3LYP-D3BJ/def2-TZVPP level of theory, including solvent effects (ethanol). Statistical mechanics contributions at 298 K were incorporated into the Gibbs free energy calculations, Δ*G* = Δ*H* – *T*Δ*S*, where Δ*S* denotes the entropic term and Δ*H* represents the enthalpy change.

**Table 1:** Energetic Properties of Stationary Points (Δ*E* + ZPE), Relative to the **1** + **2** Limit, in kcal/mol, Calculated at the B3LYP-D3BJ Level^a^

	def2-TZVPP	def2-TZVPP	VTZ	Δ*G*/def2-TZVPP
stationary point	(gas-phase)	(ethanol)	(gas-phase)	(ethanol)
**1 + 2**	0.0	0.0	0.0	0.0
**TS1**	13.4	9.7	11.7	28.6
**MIN**	–19.2	–14.3	–21.0	–5.8
**INT1**	–7.2	–9.5	–14.6	6.6
**P1**	–15.6	–11.3	–17.3	–2.9
**INT2**	–11.3	–10.7	–12.7	1.6
**P2**	–37.7	–30.5	–40.1	–25.2

a A comparison with the corresponding Gibbs free energies, in kcal/mol, for the ethanol phase is also provided.

**Figure 5:**
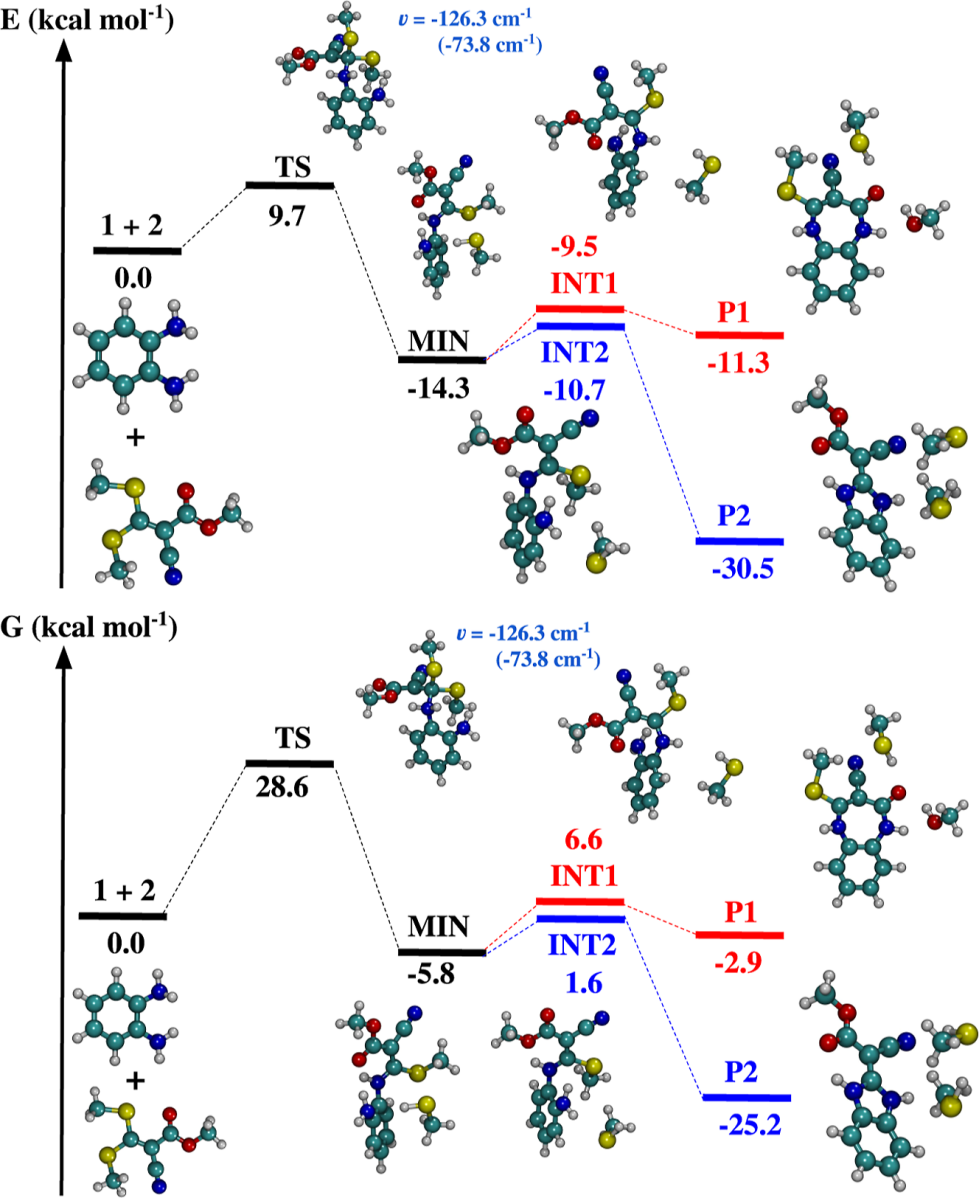
Relative energies including solvent effects (ϵ = 24.3) for the stationary points, calculated at the B3LYP-D3BJ/def2-TZVPP level of theory. Top: Δ*E* + ZPE corrections; bottom: Gibbs free energies, Δ*G*. The imaginary frequency of the **TS** is also reported.

The bimolecular reaction initiates with the association of compounds **1** and **2**, proceeding through a transition state (**TS**) characterized by a relative energy of 9.7 kcal/mol with respect to the dissociation limit. In the gas phase, this barrier increases significantly to 13.4 kcal/mol. Calculations employing the cc-pVTZ basis set indicate that the process is endothermic, with a Gibbs free energy change of 11.7 kcal/mol. The theoretical Gibbs free energy at 298 K is estimated at 28.6 kcal/mol, a value markedly elevated due to the substantial entropy contribution of the reactants (approximately −61 kcal/mol). The formation of methanethiol fragment requires cleavage of the C­(β)-S bond and a proton transfer from the C­(β)-N site, releasing 24 kcal/mol exothermically; meanwhile, the B3LYP-D3BJ/def2-TZVPP calculation in gas phase overestimates this value by 8.6 kcal/mol, while the B3LYP-D3BJ/VTZ method overestimates it by 8.7 kcal/mol.

Our B3LYP-D3BJ/def2-TZVPP (ϵ = 24.3) calculations predict a van der Waals minimum geometry located 14.3 kcal/mol below the **1** + **2** reference energy, with an associated Gibbs free energy change of Δ*G* = −5.8 kcal/mol. As illustrated in Figure 5, similar to the reaction involving 1,3-diaminopropan-2-ol and 1,1-bismethylsulfanyl-2-nitroethylene,^43^ which also features ketene dithioacetal motifs, two pathways are possible for the reaction between *o*-phenylenediamine (**1** and methyl 2-cyano-3,3-bis­(methylthio)­acrylate (**2**). The first pathway (highlighted in red) leads to the formation of product **P1** via an intermediate, denoted as **INT1**, which lies −11.3 kcal/mol below the energy of the reactants. The calculated energy of **P1** is 1.8 kcal/mol lower than that of **INT1**. Notably, this difference increases to 8.4 kcal/mol when solvent effects are not considered.

The second pathway (in blue) proceeds via a notable energy barrier of 4.4 kcal/mol relative to the **MIN** structure, leading to the formation of intermediate **INT2**. This barrier increases slightly to 7.9 and 8.3 kcal/mol when calculated using the def2-TZVPP (without solvent effects) and VTZ basis sets, respectively. Our calculations also indicate that the conversion of **INT2** to the product **P2** is exothermic by 19.8 kcal/mol. This transformation involves the synchronous cleavage of one C–S and one N–H bond, accompanied by the formation of an S–H bond. Table 1 and Figure 5 show that the decomposition of the reactants into product **P2** is exothermic, releasing approximately −30.5 kcal/mol. The calculated numbers employing the def2-TZVPP (without solvent effects) and VTZ basis sets are −37.7 kcal/mol and −40.1 kcal/mol, respectively. These values are in qualitative agreement with the computed Gibbs free energy results.

The present DFT calculations indicate that the second pathway leading to benzimidazole formation is significantly more thermodynamically favorable than the first, in agreement with the experimental findings reported by Baliza and co-workers.^22^

### Isolated Benzimidazole/Benzodiazepine

We now focus our analysis on characterizing the molecular properties of the isolated benzimidazole and benzodiazepine reaction products. The corresponding B3LYP-D3BJ reactivity descriptors are summarized in Table 2, while their ball–stick representations of the molecular structure, HOMO–LUMO molecular orbitals, and IR spectra are displayed in Figure 6.

**Table 2:** Frontier Molecular Analysis, in eV, Calculated Employing the B3LYP-D3BJ/def2-TZVPP Level of Theory with (without) Solvent Effects (ϵ = 24.3)^a^

parameter	benzodiazepine	Benzimidazole
*I*	6.16 (6.06)	5.77 (5.84)
*A*	1.94 (2.00)	1.04 (1.21)
*E*_gap_	4.21 (4.05)	4.72 (4.62)
χ	4.05 (4.03)	3.41 (3.52)
η	2.10 (2.02)	2.36 (2.31)
μ_0_	–4.05 (−4.03)	–3.41 (−3.52)
ω	3.90 (4.01)	2.45 (2.68)
*S*	0.23 (0.24)	0.21 (0.21)
Δ*N*_max_	1.92 (1.98)	1.44 (1.52)

a The Δ*N*_max_ values are in *e*.

**Figure 6:**
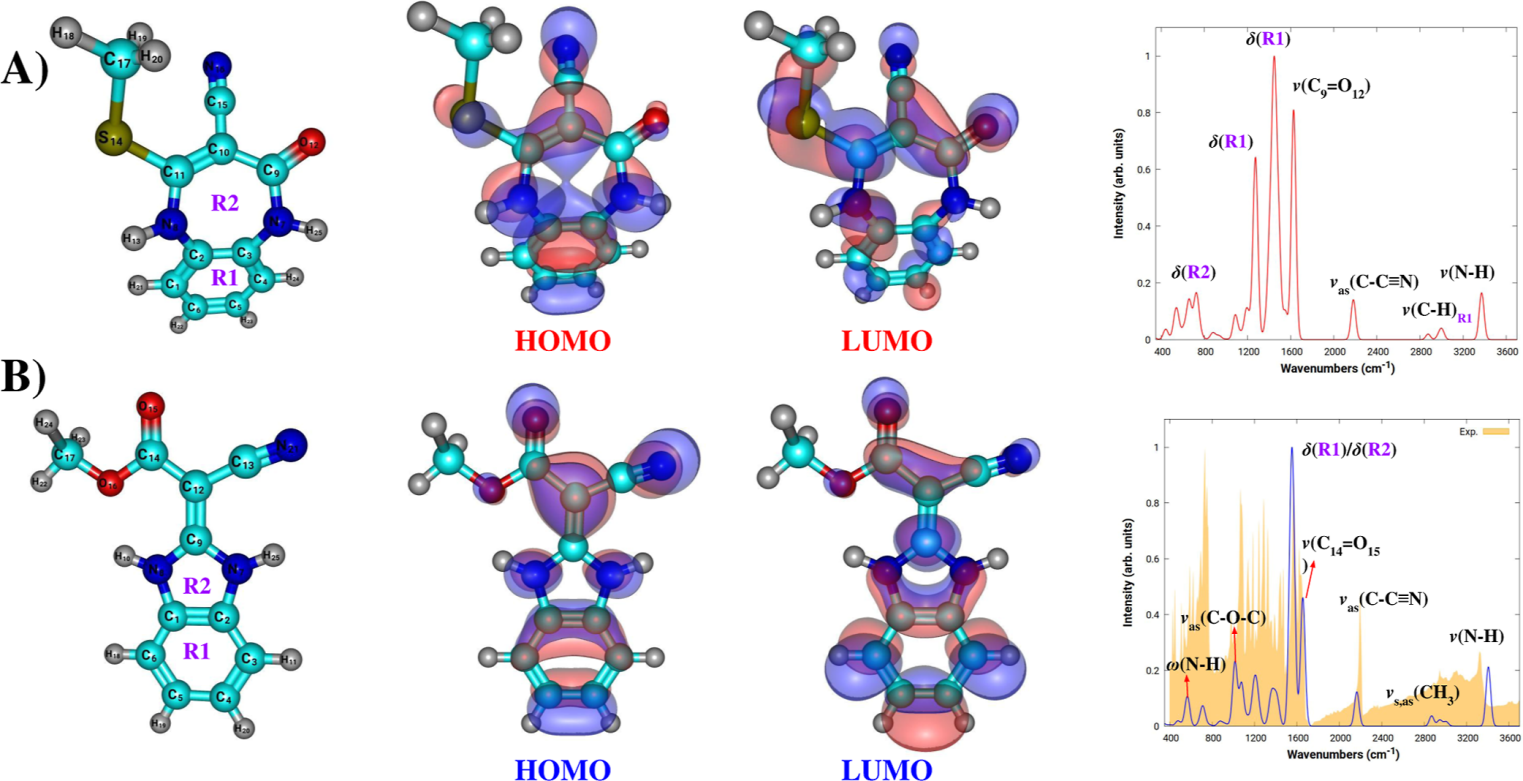
Stationary points, HOMO–LUMO molecular orbitals and IR spectrum for (A) benzodiazepine (B) benzimidazole calculated using the B3LYP-D3BJ/def2-TZVPP level of theory. Yellow shaded area shows the experimental results from Baliza and co-workers.^22^ Notation and acronym: ν = stretching, δ = deformation, ω = wagging, as = asymmetric, and s = symmetric.

The calculated HOMO–LUMO energy gaps in ethanol are 5.77 eV (benzimidazole) and 6.16 eV (benzodiazepine), indicating a 0.38 eV stability advantage for benzimidazole. Similar energy gap results were reported for the heterocyclic compounds 2-(nitromethylene)­hexahydropyrimidin-5-ol (a six-membered ring) and (2-(nitromethylene)­oxazolidin-5-yl)­methanamine (a five-membered ring), which exhibit optical energy gaps of 4.82 and 5.06 eV, respectively.^43^ Notably, the seven-membered benzodiazepine ring displays greater stability, as reflected by its comparatively higher energy gap, suggesting enhanced electronic stability over five- and six-membered species. Our computations indicate that benzimidazole is slightly less reactive than benzodiazepine. However, despite its lower energy gap, our DFT calculations suggest that the formation of benzimidazole (a five-membered ring) is thermodynamically more favorable.

Several aromatic seven-membered rings were analyzed using DFT calculations by Lin and co-workers,^46^ who reported HOMO–LUMO energy gaps ranging from 3.0 to 6.0 eV. Their values are consistent with our findings. However, theoretical values reported for nanographenes containing fused seven-five-seven (7–5–7)-membered rings are significantly lower, on the order of 2.0 eV.^47^

Elevated values of chemical potential and electrophilicity index are characteristic of strong electrophilic character, indicating a molecule’s enhanced capacity to accept electrons. The present B3LYP calculations in ethanol for benzodiazepine are μ_0_ = −4.05 eV and ω = 3.90 eV. These values differ by approximately 0.65 and 1.50 eV, respectively, from the corresponding values for benzimidazole, highlighting benzodiazepine as a strong candidate for electron-accepting species. The positive Δ*N*_max_ values are in the following order: ethanol < gas at the B3LYP level. Analysis on the seven-membered ring structures X_2_C_4_H_4_C (X = CH, N, P, and As) provided theoretical Δ*N*_max_ data in the range of 0.83–1.28*e* to the singlet states, which are in qualitative agreement with the present results.^48^

We performed quantum chemistry calculations to gain insights into the infrared (IR) spectrum of benzimidazole in the range of 400–3800 cm^–1^, including benzodiazepine for comparison. The resulting spectra are presented in Figure 6. For reference, the experimental IR spectrum of benzimidazole reported by Baliza et al.^22^ was included in Figure 6B as the yellow shaded spectrum. It is important to note that the calculated vibrational frequencies were scaled by a factor of 0.94, which was determined as the average ratio between the experimental values and the corresponding B3LYP-D3BJ frequencies. For convenience, the same scaling factor was also adopted for benzodiazepine.

Notably, both compounds exhibit similar spectral features in the higher-energy region from 2000 to 3800 cm^–1^. The current study found two weak peaks for benzodiazepine at 3365 and 3377 cm^–1^, respectively, suggesting the presence of N–H stretching vibrations. For benzimidazole, our calculations predict N–H stretching vibrations near 3410 cm^–1^. This compares reasonably well with the experimental value of 3325 cm^–1^ reported in the literature.^22^ In addition, the experimental vibrational harmonic frequency for a free imidogen (NH) molecule in its ground triplet state is 3282 cm^–1^.^44^ The methyl (−CH_3_) group exhibits symmetric and asymmetric stretching vibrations in the range of 2800–3000 cm^–1^.

The region below 1600 cm^–1^ exhibits several distinct vibrational bands. A prominent peak at 1450 cm^–1^ in the IR spectrum shown in Figure 6 A) is attributed to an aromatic deformation vibration of the **R1** ring. Additionally, a less intense peak is observed at 1628 cm^–1^, corresponding to the carbonyl stretching vibration, ν­(C=O). The vibrational harmonic frequency for a free carbon monoxide in its ground X-state is 2170 cm^–1^,^44^ which differ by ∼ 540 cm^–1^ in comparison with previous assignment. For benzimidazole, the most intense peak at 1561 cm^–1^ is attributed to coupled deformation vibrations involving both the **R1** and **R2** rings. The experimental value^22^ of 1568 cm^–1^ demonstrates good agreement with our prediction. The band at 1655 cm^–1^ is due to the ν­(C=O) vibration, which is in quantitative concordance with the experimental observation of 1622 cm^–1^ (27 cm^–1^ higher than that of benzodiazepine molecule).

NMR chemical shifts were calculated using the gauge-independent atomic orbital (GIAO) method.^49^ All ^13^C and ^1^H chemical shifts were referenced to tetramethylsilane (TMS), with the reference values computed at the same level of theory. The chemical shifts were determined using the following expression

9 δ(X)=σ(TMS)−σ(Theo.)

To validate our structural optimizations, we compared the experimental ^1^H and ^13^C NMR chemical shifts reported by Baliza et al.^22^ and Misra et al.^23^ with our DFT values in Table 3. This benchmarking confirms the accuracy of our computational methodology.

For benzimidazole, the experimental δ 12.34 ppm (s, 2H) taken from^22^ corresponds to two N–H protons of the benzimidazole ring. The high chemical shift is typical for N–H groups where free electrons on nitrogen resonates with double bonds. These hydrogen atoms are labeled as H_10_ and H_25_ in Figure 6 (structural view) and Table 3. The calculated ^1^H NMR chemical shifts of 10.90 ppm differ by about 1.44 ppm from the experimental measurement. Baliza et al. observed a singlet at 3.66 ppm corresponding to the three protons of the methoxy (−OCH_3_) group, while the DFT prediction yields a value of 3.55 ppm, representing an error of approximately 3%. The remaining ^1^H NMR signals in the range of 7.17–7.40 ppm are assigned to aromatic protons of the benzimidazole **R1** ring. These values are in qualitative agreement with our findings.

**Table 3:** Theoretically Calculated and Experimentally Determined Chemical Shifts (in ppm) for ^13^C and ^1^H NMR Spectrum of

	benzimidazole			benzodiazepine	
atom number	Theo.	Exp.^22^	atom number	Theo.	Exp^23^
C_1_	134.41	131.37	C_1_	124.87	125.45
C_2_	133.43	131.37	C_2_	132.55	127.40
C_3_	121.29	123.28	C_3_	131.39	127.40
C_4_	124.60	119.75	C_4_	123.78	125.45
C_5_	124.60	119.75	C_5_	122.64	117.40
C_6_	121.29	123.28	C_6_	122.64	117.40
C_9_	168.39	167.66	C_9_	165.30	161.70
C_12_	52.44	50.95	C_10_	79.15	65.39
C_13_	112.45	111.89	C_11_	160.27	137.40
C_14_	151.55	152.91	C_15_	107.33	111.90
C_17_	52.44	51.47	C_17_	17.93	15.64
H_10_	10.90	12.34	H_13_	6.24	7.48
H_11_	6.93	7.40	H_18_	1.86	1.77
H_18_	6.93	7.40	H_19_	1.86	1.77
H_19_	7.04	7.17	H_20_	1.86	1.77
H_20_	7.04	7.17	H_21_	6.96	6.75
H_22_	3.55	3.66	H_22_	7.30	7.23
H_23_	3.55	3.66	H_23_	7.48	7.23
H_24_	3.55	3.66	H_24_	7.00	6.75
H_25_	10.90	12.34	H_25_	5.63	4.14

Concerning the ^13^C NMR chemical shifts, the computed values show good agreement with experimental data, supporting the proposed structural assignments. The experimental resonance^22^ at δ 167.66 ppm is attributed to the ester carbonyl carbon (C=O), differing by only 0.73 ppm from the DFT value. The experimental ^13^C NMR chemical shifts ranging from 119 to 132 ppm are attributed to the aromatic carbons of the benzimidazole **R1** ring. These data agree well with our numerical interval of [121,135] ppm. Additionally, a comparison between experimental and theoretical data is presented in Figure S8 of the Supporting Information using a linear regression model, from which the following equation was derived for ^1^H NMR chemical shifts

10 y=1.003x−1.559852

with correlation coefficient of 0.99. For ^13^C, we have

11 y=1.773x−0.671409

Analysis of the results for benzodiazepine indicates that the experimental signal^23^ at δ 7.48 ppm (s, 1H) corresponds to the NH proton of the diazepine ring, designated in this work as H_13_. Our DFT result of 6.24 ppm differs by only 1.24 ppm from the experimental data. The ^1^H NMR signals observed by Misra et al. in the range of 6.75–7.23 ppm appear as a multiplet corresponding to the aromatic protons of the benzene ring. Our theoretical calculations yield chemical shift values ranging from 6.96 to 7.48 ppm, which are in good agreement with the experimental data. Meanwhile, the experimental signal at 1.77 ppm (s, 3H) is assigned to a methyl group (−CH_3_) connected to sulfur (−S–CH_3_). This value deviates by approximately 5% from our predicted chemical shift by DFT calculations.

The experimental ^13^C NMR chemical shift at 161.70 ppm corresponds to the carbonyl carbon (C=O) of the diazepine ring. Our calculated δ of 165.30 ppm deviates from the experimental value by only 2%. The carbonitrile group (C_15_≡N_16_) is experimentally assigned around 111.90 ppm, while the corresponding theoretical value is 107.33 ppm. To conclude this part, a comparison between experimental and theoretical data is presented in Figure S9 of the Supporting Information using a linear regression model. For such, we derive the following analytical representations for ^1^H and ^13^C NMR chemical shifts, respectively

12 y=0.961x−0.4202

13 y=1.029x−0.3530

In both cases, a highly significant positive correlation (*R* = 0.96) was observed between the theoretical and experimental data, demonstrating the robustness and predictive power of our methodology for accurately calculating NMR parameters in this class of species.

The two reaction pathways discussed in the literature,^22,23^ one of which was developed by us, were modeled theoretically. Although the reaction leading to product **P1** (benzodiazepine) was experimentally observed by Misra et al.,^23^ our experiments under different conditions yielded only product **P2** (benzimidazole). Theoretical calculations indicate that product **P2** is the more favorable outcome, which is consistent with the recent findings reported by Baliza and co-workers.^22^

## Conclusion

Synthesis of N-heterocyclic compounds remains extremely important to developments in biological and material sciences, motivating continued theoretical and experimental investigations. In this work high-level DFT calculations were performed to provide a plausible reaction mechanism between methyl 2-cyano-3,3-bis­(methylthio)­acrylate and *o*-phenylenediamine. Structural and energetic information on key energy minima and transition states relevant to potential reaction pathways is reported for both the gas phase and ethanol solution. These data were then used to construct a realistic reaction model. The nature of the stationary points was analyzed using the Global Optimizer Algorithm for Transition states (GOAT), as implemented in the ORCA package. The interaction between reactants reveals a transition state with an energy barrier of 9.7 kcal/mol, followed by the formation of a van der Waals minimum located 14.3 kcal/mol below the energy level of the reactants. The present findings indicate that two reaction pathways originate in this step. The first pathway, initiated by the formation of intermediate **INT1**, yields benzodiazepine (denoted as product **P1**). The second pathway leads to the formation of benzimidazole (**P2**). As shown in Figure 5, both reaction pathways are exothermic; however, the production of benzimidazole is thermodynamically more favorable, exhibiting an energy difference of −19.2 kcal/mol relative to the first pathway when solvent effects are considered. Theoretical results for isolated benzimidazole and benzodiazepine show excellent agreement with experimental NMR and IR spectroscopic data. This strong correlation between computational and experimental results not only validates the proposed reaction mechanism but also demonstrates the predictive capability of density functional theory in the rational design and mechanistic understanding of complex heterocyclic systems.

## Supplementary Material


