# Supplementary material for: Dual Pathways, One Framework: Theoretical Insights into the Benzimidazole × Benzodiazepine Crossroads from o‑Phenylenediamine and 2‑Cyanoacrylate Derivatives
Source: ACS Omega. 2025 Oct 21;10(43):51132–42. doi: 10.1021/acsomega.5c05925 (PMC12593153; doi:10.1021/acsomega.5c05925)
Supplement: Supplementary file 1 [file ao5c05925_si_001.pdf]

Dual Pathways, One Framework: Theoretical Insights into the Benzimidazole × Benzodiazepine Crossroads from o-Phenylenediamine and 2-Cyanoacrylate Derivatives

Supplementary Material

Ramon S. da Silva[a] Ana J. F. Souza[c], Diego P. Sangi[c], and Rodrigo G. Amorim[b]

[a]Departamento de Física, Universidade Federal de Juiz de Fora, Juiz de Fora, 36036-330, Minas Gerais, Brazil

[b]Departamento de Física - Instituto de Ciências Exatas - ICEx, Universidade Federal Fluminense, Volta Redonda, 27213-145, Rio de janeiro, Brazil

[c]Departamento de Química - Instituto de Ciências Exatas - ICEx, Universidade Federal Fluminense, Volta Redonda, 27213-145, Rio de janeiro, Brazil

E-mail: ramon.sousa@ufjf.br

—--

Keywords:

DFT calculation; Reaction mechanism; Benzimidazol; Benzodiazepine

—--

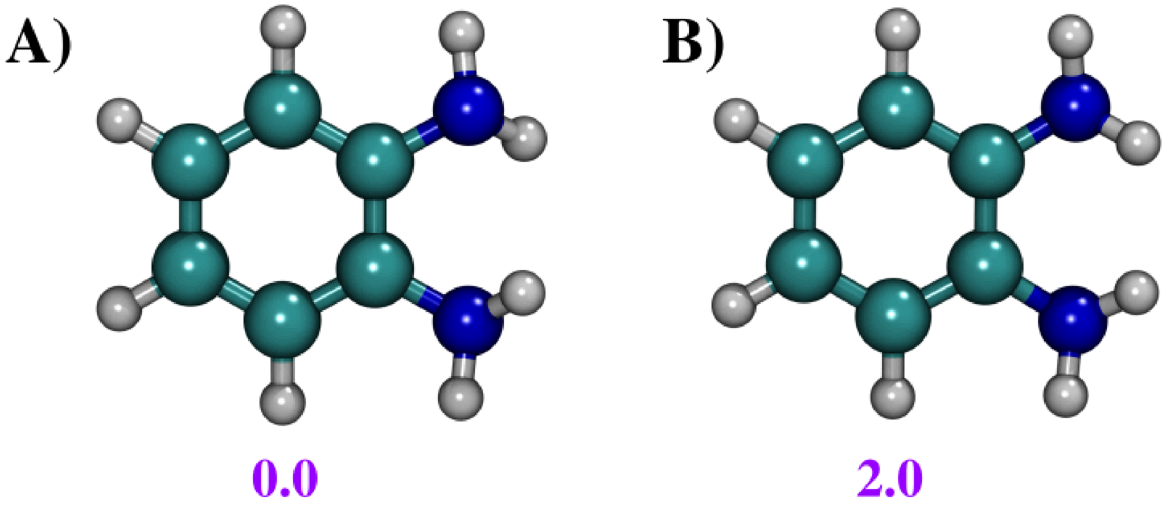

Figure S1: Minimum structures for ortho-phenylenediamine (compound **1**, *reactants*) using GFN2-XTB in gas phase. Energy differences from the global minima in kcal/mol were collected and are shown below each structure.

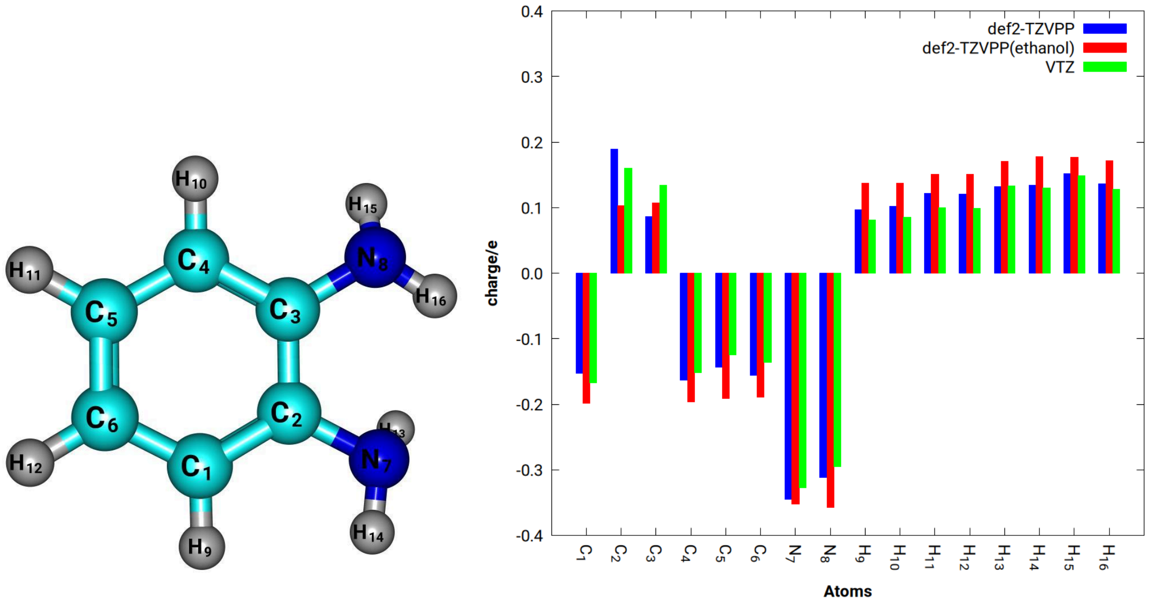

Figure S2: Geometry equilibrium calculated using the B3LYP-D3BJ/def2-TZVPP level of theory for o-phenylenediamine (compound **1**, *reactants*) together with the corresponding atomic charge values obtained from Mulliken population analysis.

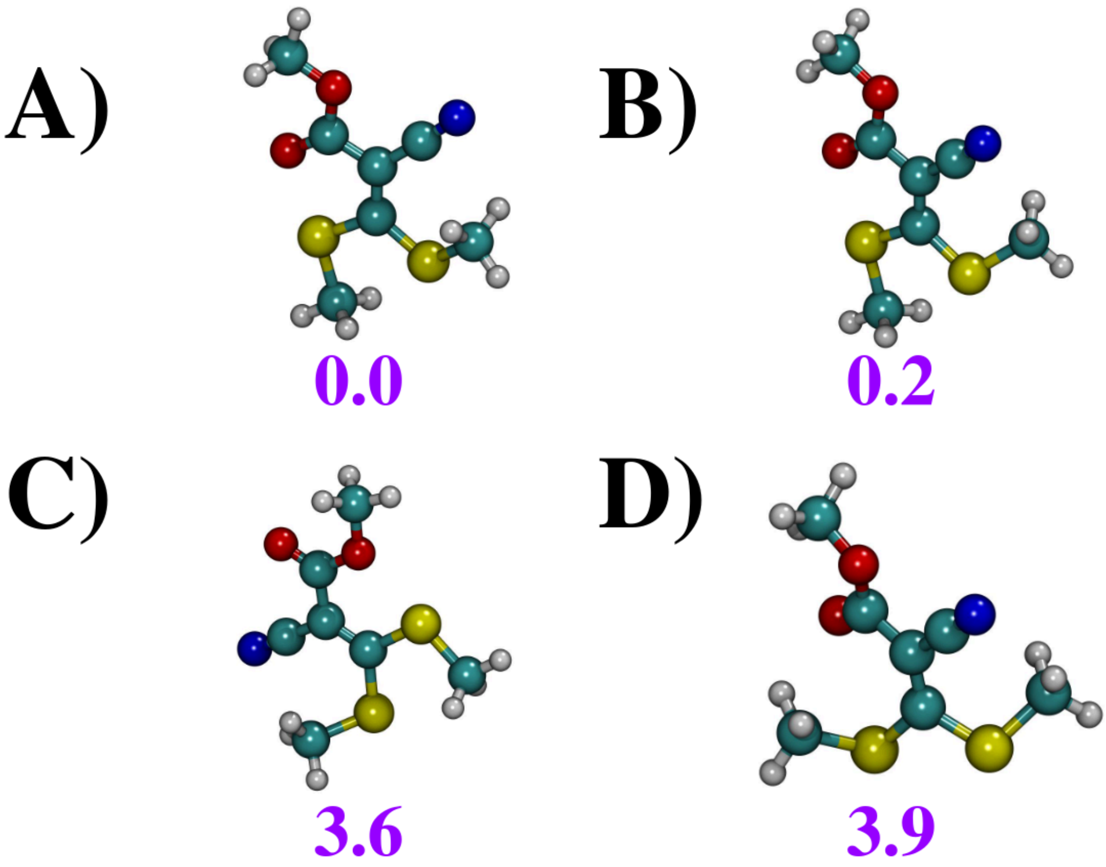

Figure S3: Minimum structures for methyl 2-cyano-3,3-bis(methylthio)acrylate (compound **2**, *reactants*) using GFN2-XTB in gas phase. Energy differences from the global minima in kcal/mol were collected and are shown below each structure.

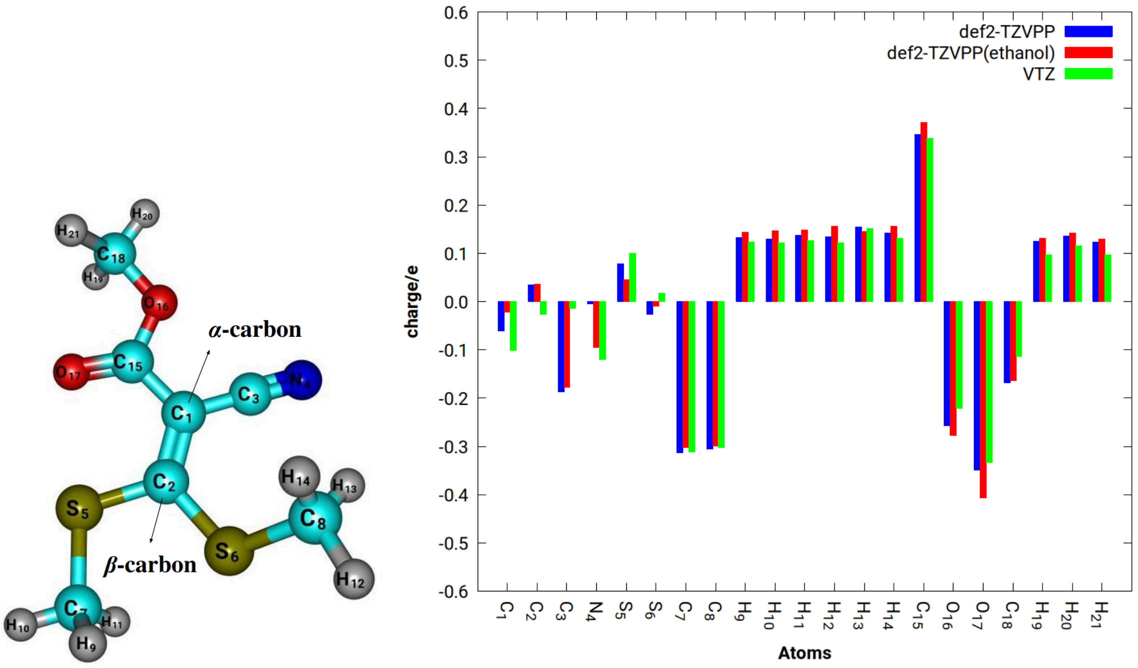

Figure S4: Geometry equilibrium calculated using the B3LYP-D3BJ/def2-TZVPP level of theory for methyl 2-cyano-3,3-bis(methylthio)acrylate (compound **2**, *reactants*) together with the corresponding atomic charge values obtained from Mulliken population analysis.

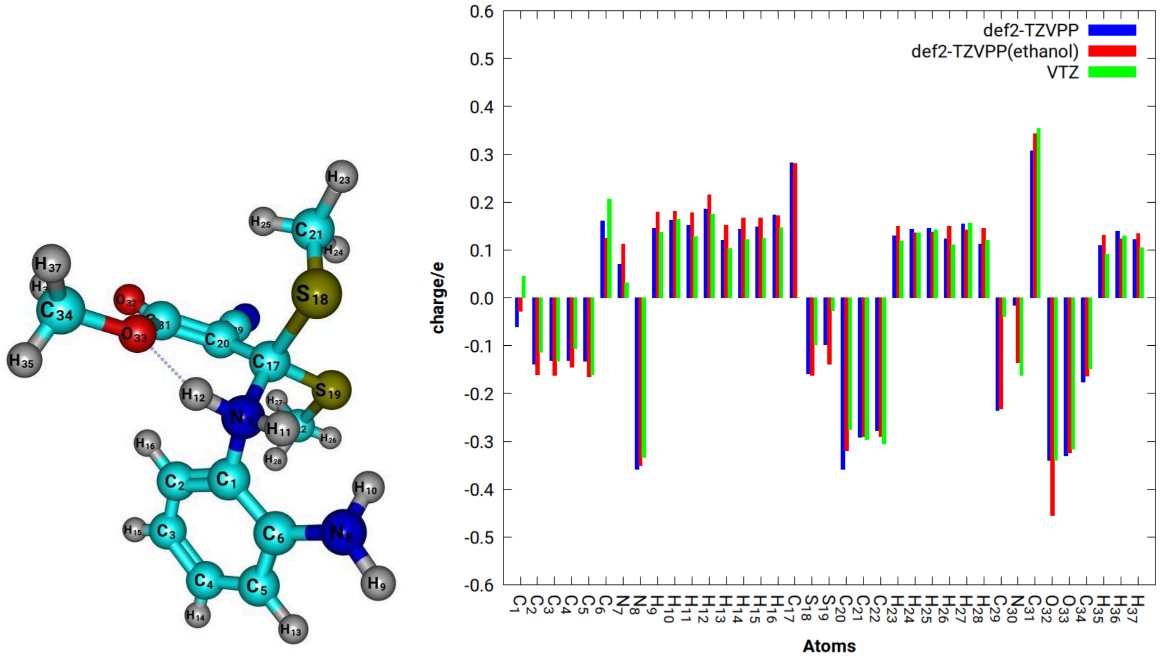

Figure S5: Geometry equilibrium calculated using the B3LYP-D3BJ/def2-TZVPP level of theory for the transition state (**TS**) together with the corresponding atomic charge values obtained from Mulliken population analysis.

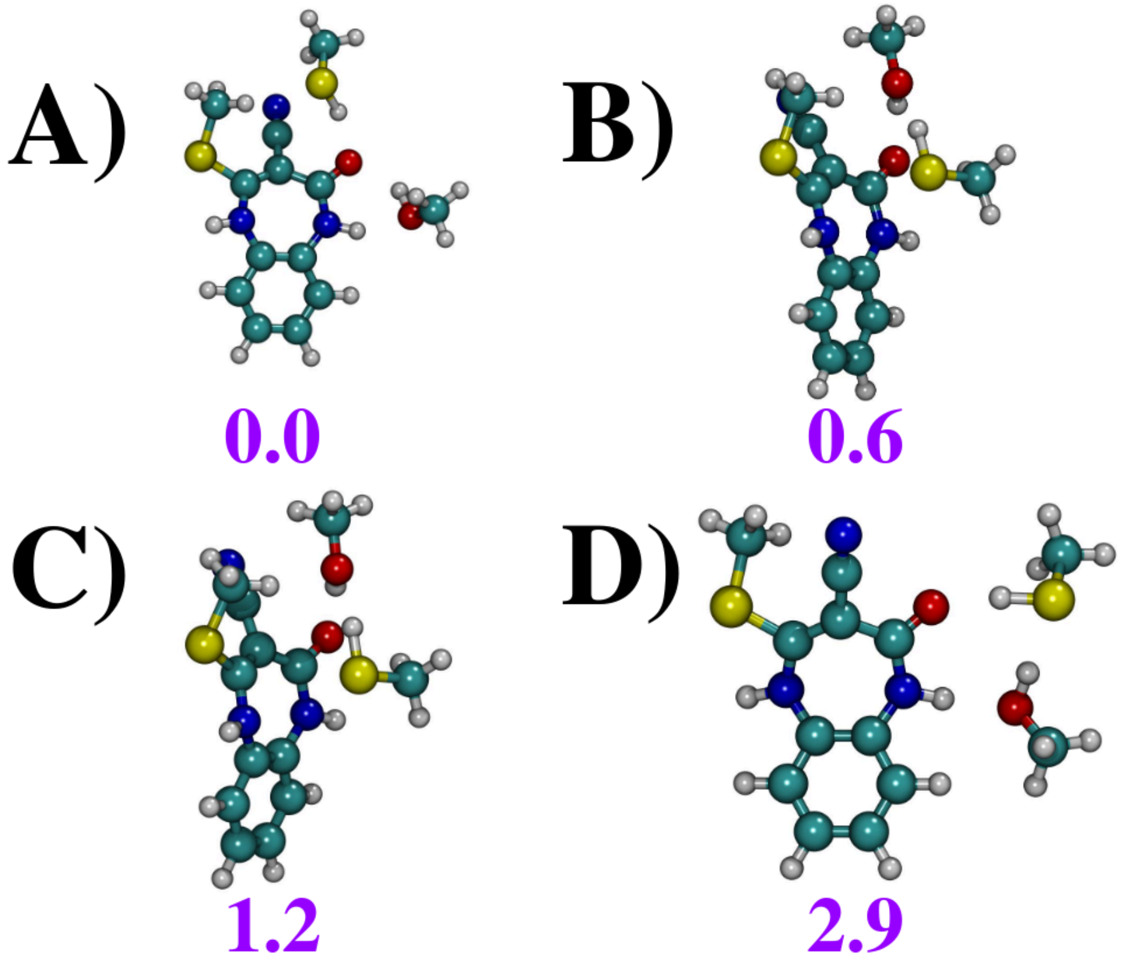

Figure S6: Minimum structures for product **P1** using GFN2-XTB in gas phase. Energy differences from the global minima in kcal/mol were collected and are shown below each structure.

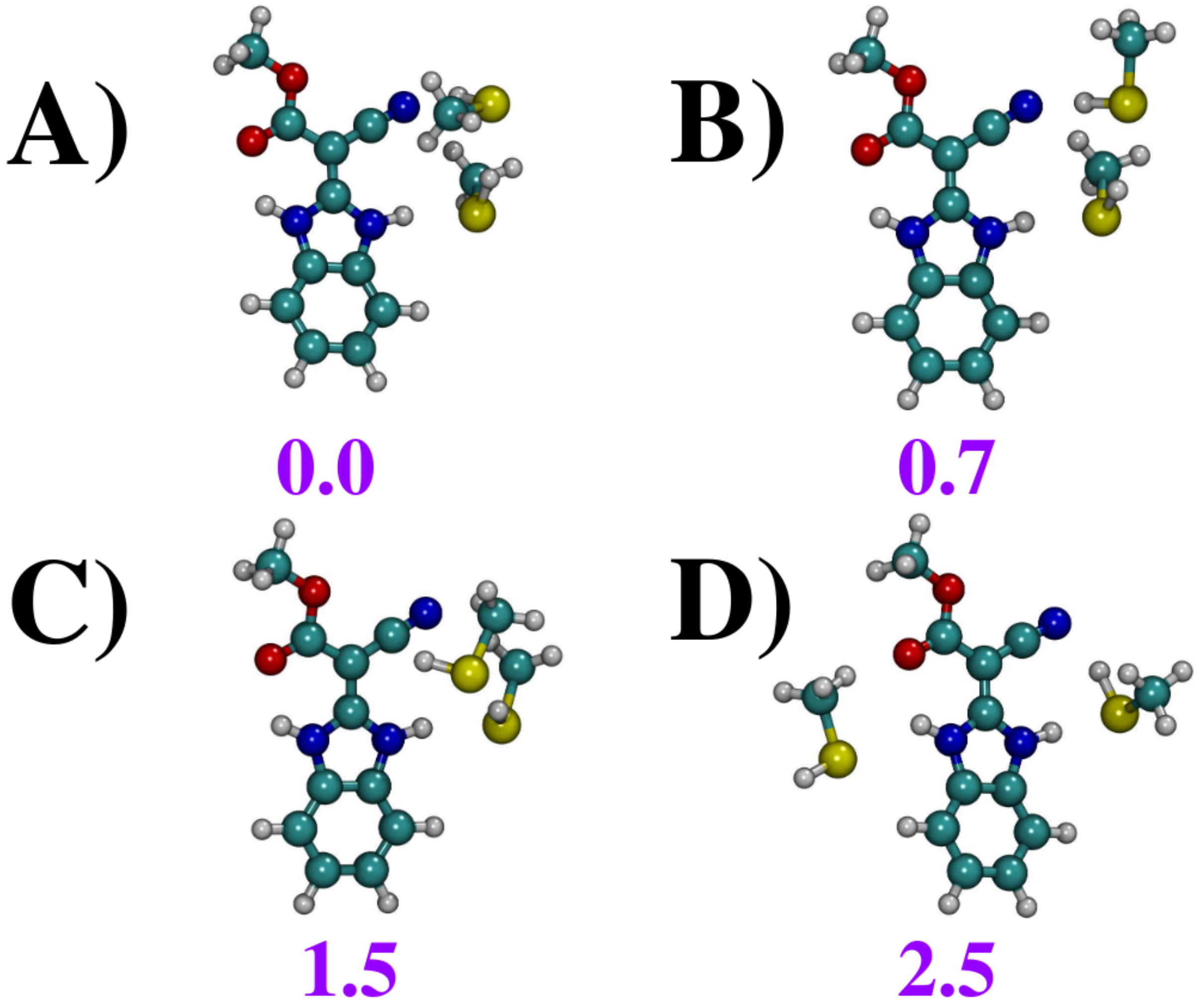

Figure S7: Minimum structures for product **P1** using GFN2-XTB in gas phase. Energy differences from the global minima in kcal/mol were collected and are shown below each structure.

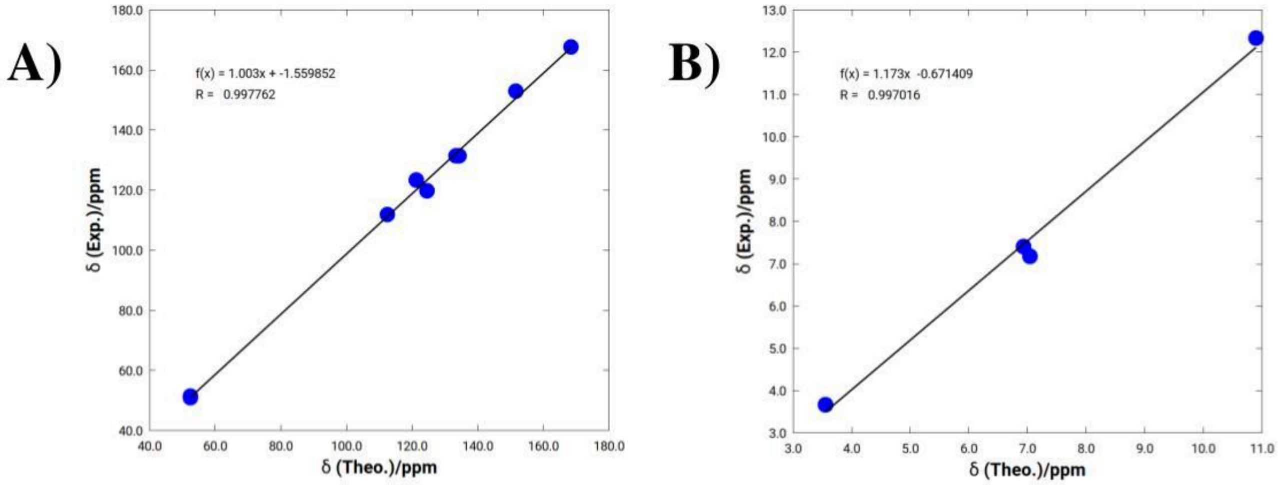

Figure S8: Comparison between theoretically calculated and experimentally determined A) ^{13}C and B) ^{1}H chemical shifts for benzimidazole (product **P2**). The R parameter of 0.99 corresponds to linear correlation between theoretical and experimental results taken from [1].

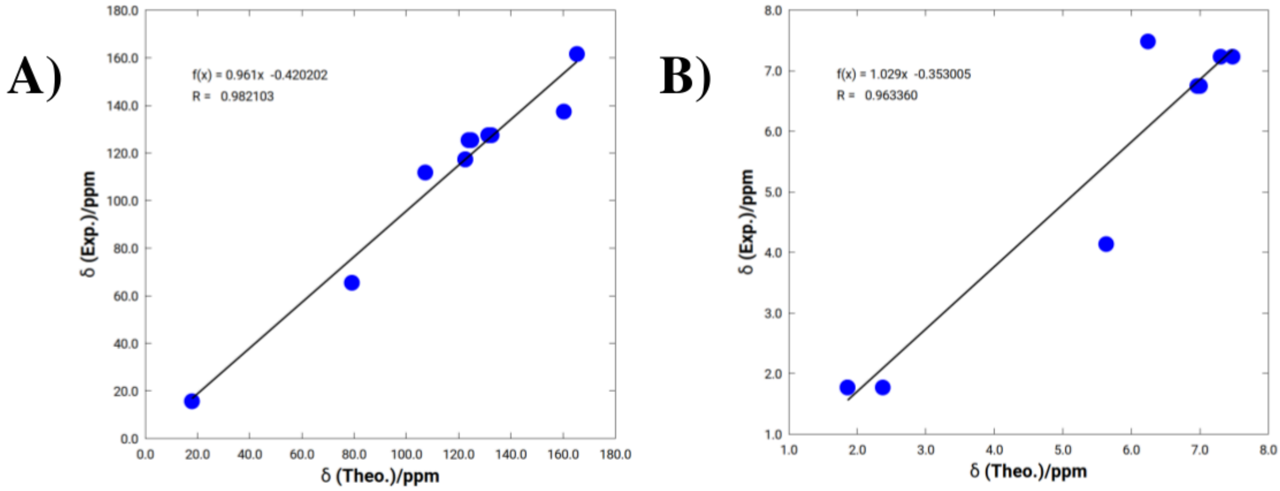

Figure S9: Comparison between theoretically calculated and experimentally determined A) ^{13}C and B) ^{1}H chemical shifts for benzodiazepine (product **P1**). The R parameter corresponds to linear correlation between theoretical and experimental results taken from [2].

Cartesian coordinates for o-phenylenediamine

C	-9.03552208785002	4.52894904324627	0.37314404308228
C	-9.43863490960764	4.06833181820401	-0.88107158144461
C	-9.52895010864272	2.67593415767952	-1.09978175470792
C	-9.21874551053404	1.80715351825197	-0.05307558676149
C	-8.83454241515431	2.28083115735370	1.19781363978792
C	-8.74104960854217	3.65085169022663	1.41149122448533
N	-9.82454836126226	4.97263694393417	-1.88074751005984
N	-10.00014665850159	2.18000052108199	-2.32594325973557
H	-8.96360356125154	5.59887091524345	0.53024652748631
H	-9.28805798009240	0.74043729750404	-0.23167557184449
H	-8.60496262084988	1.58168584003451	1.99084533879315
H	-8.43670275167373	4.03952516071707	2.37418892311627
H	-9.60947900592696	4.69914758243899	-2.82808652501690
H	-9.49234581001060	5.91192090761540	-1.71526872521728
H	-9.82436637837404	1.19191887986716	-2.44154936797396
H	-9.67354223172584	2.67340456660101	-3.14482981398918

Cartesian coordinates for methyl 2-cyano-3,3-bis(methylthio)acrylate

C	-3.94384282503359	3.40349431249621	0.00974050149745
C	-3.90478453648552	2.12860209297559	-0.53894985702081
C	-2.75412206276565	4.07453747568242	0.37820939492495
N	-1.79273601804633	4.65865833628611	0.64363944606279
S	-5.29869022058813	1.48439565951140	-1.36297716834269
S	-2.50131221263367	1.09476125131674	-0.50530174806556
C	-4.80658067366631	-0.18172136230448	-1.88198341165275
C	-1.60947191792699	1.46449996589118	1.03363696133681
H	-4.54560563924676	-0.80167025094062	-1.02883200142077
H	-5.70642898831330	-0.56950043748713	-2.35746700003182
H	-3.99792649619665	-0.15980277059389	-2.60719900352681
H	-1.08714573904235	0.53547827033635	1.25444573067090
H	-0.90124514028826	2.27540527267793	0.91351227137490
H	-2.31901071019778	1.68219633613410	1.82731160708930
C	-5.18625611867546	4.18132239247216	0.12051775511836
O	-4.98699379793080	5.35326453224206	0.74147593130706
O	-6.27117645867090	3.82126882406122	-0.29345861131262
C	-6.13876506653507	6.20785953360004	0.88267513543079
H	-6.54007235634247	6.46296349355286	-0.09614268840321
H	-5.77654548827637	7.09598178484114	1.38972980151057
H	-6.90398754494194	5.71320529758980	1.47741696932848

Cartesian coordinates for transition state (TS)

C	-1.42853241528036	-0.07687807512522	0.42908862587741
C	-1.54644630959375	1.30242418842378	0.34505519149352
C	-2.75794156192381	1.86650453486444	-0.02658615919370
C	-3.84405252166463	1.03482607912450	-0.28977788686027
C	-3.72024414467955	-0.34236060796823	-0.19834897432038
C	-2.50182487393706	-0.93388499684098	0.15404532702317
N	-0.17025906864451	-0.66387618967165	0.87983376979789
N	-2.36560823941823	-2.31281516577912	0.29740166032349
H	-3.20351377583450	-2.82551561795049	0.06355846082068
H	-1.56790283986040	-2.70253757326943	-0.19214721453533
H	-0.37364859026545	-1.56891071047441	1.31411754490824
H	0.26253913732620	-0.06374951623663	1.59963904968278
H	-4.56531615437766	-0.98412393672386	-0.41133341188053
H	-4.79615240743312	1.46305430654894	-0.57343903783382
H	-2.85463025609411	2.94022525244269	-0.09813819318994
H	-0.70075101614576	1.93039183878462	0.58065336214462
C	0.97366878041196	-0.85238209099483	-0.17093699727660
S	2.08454163963747	-2.07293875516294	0.73566209414134
S	0.38847616588275	-1.82627080798832	-1.59120743525447
C	1.58913548085507	0.45230263864628	-0.50811217057241
C	3.65175502957252	-1.76812699430084	-0.11006835568224
C	-0.60574932026388	-0.69619363165887	-2.63109715266869
H	4.36199693914397	-2.45924702280437	0.34080064931812
H	3.56390527283484	-1.97898495953125	-1.17286245897096
H	3.98262786078534	-0.74608721250264	0.05169532712562
H	-0.21333463311966	-0.74347622142792	-3.64316989829524
H	-0.52570294513377	0.32008632582512	-2.26103264997312
H	-1.64425263901915	-1.01348811973109	-2.60693113170660
C	2.03589306979063	0.68052768300912	-1.81435629538422
N	2.41735740497500	0.88722618814903	-2.89224744344347
C	1.99565553716960	1.43352940739089	0.44888505461557
O	2.60182726525539	2.46940314998650	0.20758605043311
O	1.61286635054356	1.11213915760014	1.73440297211325
C	1.91490392337359	2.02497346403181	2.80348358817240
H	0.97589600558706	2.31260054620368	3.27161631989150
H	2.42704584267038	2.89866281807020	2.41446588475494
H	2.54577201216578	1.50296162044526	3.51980291867392

Cartesian coordinates for minimum structure (MIN)

C	-0.68000998082302	0.39971047173585	-0.74486094119262
C	-1.14286089528384	1.41921634254259	-1.56689121017405
C	-1.70209513541283	1.12836579119036	-2.80370968381063
C	-1.81119753917033	-0.20392265057191	-3.19857094103564
C	-1.35939935937720	-1.22607047685734	-2.37996439620270
C	-0.77680279385303	-0.94621239884694	-1.13654942205806
N	-0.13089510023380	0.69929487018680	0.53786969345564
N	-0.23790386633368	-1.95487341800657	-0.35986317870789
H	-0.58925533786838	-2.88342622628159	-0.53383025749277
H	-0.10493793681331	-1.75981730805176	0.61963616568064
H	-0.67498321099469	0.48190603037905	1.37423461228213
H	-1.43696537537115	-2.25781951323048	-2.69848199020965
H	-2.25115585442293	-0.44946623016262	-4.15622566348257
H	-2.05511526627450	1.92488685325011	-3.44322499952181
H	-1.05560817357827	2.44138096330851	-1.22321447437732
C	1.11094342276906	1.11887576168398	0.78824996891321
S	2.06382453542973	1.52391918602123	-0.63259072679490
C	1.57400684686608	1.24652721707108	2.10861445154690
C	3.75361326538651	0.98827508719438	-0.22860421354644
H	3.71976590441511	0.08064669413365	0.36680871834939
H	4.31858717862246	1.76421298423590	0.27405744142139
H	4.19456209766410	0.76292987729036	-1.19719222012497
C	2.74783750478770	1.96958372466200	2.39861569318238
N	3.69308910212807	2.58428246800901	2.65906672144919
C	0.80296934379949	0.73781154690243	3.23499015336760
O	-0.31176542150894	0.22989503606752	3.15800166399037
O	1.44421995660757	0.88168405268122	4.40318100920234
C	0.74543106547329	0.44259777028993	5.58412140300211
H	0.52647174754665	-0.62130043695836	5.52108982848112
H	-0.17967495070248	1.00294620087819	5.70419933642322
H	1.42178020741469	0.64363783827757	6.40836567232976
C	2.01581759214278	-0.85109851644820	-3.61972875037702
S	2.71777423599953	-2.08543822791634	-2.47410361697098
H	2.76829240537461	-0.68236892211882	-4.38706892732204
H	1.81733494702631	0.07984330025447	-3.09674787954239
H	1.10468226185158	-1.22170306513488	-4.07986719251354
H	1.72752258512454	-2.08271268532892	-1.56061186783540

Cartesian coordinates for intermediate 1 (INT1)

C	1.29200958347505	-2.71880516743552	-0.16795947232087
C	0.84942605067878	-1.54995587387237	-0.78763357296048
C	-0.37548387892683	-1.53590271184462	-1.48114313798952
C	-1.15055489819009	-2.70619995302565	-1.45492339798239
C	-0.71263087437814	-3.85266068753544	-0.81957967470437
C	0.52834577745791	-3.87201029780884	-0.18204191931829
N	-0.84660568376394	-0.42397498809771	-2.15405150222979
N	1.72748911838767	-0.43895220093107	-0.76152050866636
C	-0.52352817763608	0.72024694630946	0.89303962056767
C	0.31074713679646	1.42310156757802	-0.08443816928983
C	1.47062125325690	0.87611496273236	-0.62913607878242
O	-0.20509595204393	-0.28160876509127	1.50260324120118
O	-1.69955711197285	1.35014333390681	1.08142691837248
S	2.75734227579621	1.84865168583004	-1.32884977360833
C	-0.09715191247428	2.71244305837983	-0.48610209084635
N	-0.44101391486721	3.76229734909656	-0.83048044212773
C	2.89386190161702	3.28679288287228	-0.22802790746676
H	2.18936533729284	4.06490852219023	-0.50210516642167
H	3.91332127229777	3.64441795823937	-0.35768620370455
H	2.74364633918894	2.97432937497931	0.80184974006417
C	-2.56785337689068	0.79364993161155	2.08451479610799
H	-2.85872948013904	-0.21933322233738	1.81281378914010
H	-3.43622479861354	1.44412973256726	2.10819473423246
H	-2.07211044397035	0.78506544937759	3.05349791779843
H	-0.16484791816395	0.24828038252902	-2.46620411235946
H	-1.53420174611096	-0.62229581286962	-2.86463732191998
H	2.70650652877246	-0.67424054227906	-0.85909760557379
H	2.24957001579524	-2.70007198518574	0.33630196348296
H	0.88750378399152	-4.76782443161240	0.30446559460369
H	-1.33170263787816	-4.73974520584815	-0.83370254727547
H	-2.10232987702782	-2.69985393574771	-1.97105736392872
C	1.45297338913106	-3.22184161234856	-3.97398649020742
S	2.63657016203972	-1.85102013182029	-4.19448891441902
H	0.44124938328653	-2.89957690014417	-4.20237291274006
H	1.49940206349627	-3.61726668398015	-2.96363574642342
H	1.75098055720280	-3.99438504539439	-4.67967672099682
H	2.09189075308660	-0.99624698298944	-3.31786956130698

Cartesian coordinates for product 1 (P1)

C	-0.91913222322329	-1.82248882144893	-3.29872794846265
C	-0.40693219773292	-1.39583851874225	-2.07582511426486
C	-1.06628461087311	-1.71448346707155	-0.89157207769167
C	-2.24003860674545	-2.46551789333798	-0.95083185976225
C	-2.75707961783997	-2.87832103211867	-2.17049154562152
C	-2.09468347305901	-2.55679123014072	-3.35105058080299
N	-0.62736437631348	-1.24805918210655	0.36367922361199
N	0.75703163531012	-0.59241101009612	-2.08555210067596
C	0.63419045487199	-1.29130183722459	0.87452509481996
C	1.80469602453674	-1.26282773455412	-0.01168644887830
C	1.82996482409873	-0.70736927461157	-1.28446443196161
O	0.79604563048425	-1.29300839979291	2.10369541566257
O	-1.72365586812689	-0.56352962798152	3.06351383640923
S	3.28420181938255	-0.10015202128218	-2.06329427429648
C	3.01245360609731	-1.65744896642946	0.60482190261416
N	3.98925139539163	-2.01693961632121	1.10850619532563
C	4.20587541991728	0.75680164855073	-0.75222280855525
H	4.77001688396310	1.52914815507191	-1.27085558742086
H	4.87980219252761	0.08257881284784	-0.23556280687118
H	3.50767803100782	1.21595669783714	-0.05675248110597
C	-1.74259584408644	0.86280855268689	3.00207053785693
H	-1.27167330419208	1.30788760217531	3.88225546149799
H	-2.78428046099511	1.17664734221278	2.96366409999742
H	-1.23166652038984	1.23179232425167	2.10784720841943
H	-1.33737376239291	-1.21269534651262	1.09047588816464
H	0.89125111414381	-0.04171409343286	-2.92190654753699
H	-0.39027501048048	-1.55994495062447	-4.20567022075114
H	-2.48796464047854	-2.87463732245755	-4.30643192684345
H	-3.67498546501103	-3.44916838812339	-2.19603945557364
H	-2.75114926390501	-2.70532808347650	-0.02762918898560
H	-0.79517022454219	-0.85122129918814	3.01624893666723
C	3.80302161798828	1.37344708577793	2.99871742910605
S	2.15923320171621	1.92826833196756	2.42952384704165
H	3.76043227523589	1.04256845611159	4.03313592604746
H	4.46402243111436	2.23386307732397	2.92248671860704
H	4.17693641240886	0.57458968388736	2.36472697942767
H	1.54930050489683	0.73274033125451	2.49147269541291

Cartesian coordinates for intermediate 2 (INT2)

C	-1.97913803242034	-0.78013700165602	0.23429737470343
C	-1.29340825917683	0.26191266611547	-0.41548406745606
C	-1.73388309595970	0.75103301713115	-1.64122039132632
C	-2.82995618844445	0.19204690805358	-2.27867536897448
C	-3.48995822006018	-0.87419192278777	-1.66810862281917
C	-3.07612264267024	-1.34764257131660	-0.43572891291776
N	-0.16738090582536	0.88765906819263	0.20071009656572
N	-1.65539510276546	-1.20868110522003	1.50726644299376
C	1.00970823673652	0.33982848779425	0.51307278671065
S	1.49145673815007	-1.23089372651018	-0.11912206663729
C	0.86497301759844	-1.30934097905138	-1.82518978051884
C	1.91694041599893	0.94138615471703	1.40194606760240
C	3.19747385598233	0.36590502863496	1.57948991650392
C	1.69471394710987	2.13599907843086	2.21768370036757
O	2.49379147623512	2.55775017713985	3.03291705389675
O	0.51848954302052	2.75216524237035	1.97681477724844
C	0.22789160486378	3.93034866362214	2.75693912837232
H	1.50642149886312	-2.04937212558131	-2.30035597074219
H	-0.16878975971736	-1.63174027407794	-1.86686169568504
H	0.99453332761184	-0.34951478198637	-2.31501400349412
N	4.25113495044820	-0.07746115489869	1.75720424739741
H	-0.74766775771968	4.26290894952584	2.41815600590885
H	0.97943737142398	4.69464703468081	2.57338323445604
H	0.20269847740169	3.68123820913407	3.81529262836493
H	-0.31066504799906	1.82393872878994	0.56250691406060
H	-0.72744278154110	-1.00034753568071	1.83645282733787
H	-1.92006102395842	-2.16503259552385	1.71068297922396
H	-1.18162125566167	1.56154146330607	-2.09798867364951
H	-3.60797009073532	-2.15958209703571	0.04240568872888
H	-4.33983623300936	-1.33542395941164	-2.15396508196203
H	-3.15743159347119	0.57177142800990	-3.23607483542502
C	-1.00866971812808	-4.33577454382964	-0.28886520254947
S	-2.04325514072886	-4.70325532929944	1.16879822401065
H	-0.45284126734490	-5.21668263615396	-0.59773029555973
H	-0.33153782541769	-3.51406634414598	-0.07430988922976
H	-1.69219745844965	-4.04104769028001	-1.08104685990068
H	-1.04883506023923	-4.98989193120153	2.02342162439321

Cartesian coordinates for product 2 (P2)

C	-0.01811499532700	-0.52250072601594	-2.09386081621122
C	-1.33509475947622	-0.57178456720161	-1.61423407056198
C	-2.42282693843830	-0.46848201616451	-2.46731539347556
C	-2.14709521008642	-0.31468392587015	-3.82383662274983
C	-0.83290788054635	-0.26814886020981	-4.30276991514755
C	0.25845381040676	-0.37184329088823	-3.44366269135213
N	-1.24649669211427	-0.73246169299048	-0.23585293922646
N	0.80303358604019	-0.64913081364051	-0.98177908765846
C	0.05433014655465	-0.76977612096093	0.13873580543709
S	-4.28316091530558	-0.28892682824173	1.13616534266892
C	-4.49147016768929	-1.56567818296142	2.42347578869344
C	0.55759366455300	-0.90666056291747	1.44826775836509
C	-0.33568387448445	-1.04857708690805	2.51878898471322
C	1.98182477084677	-0.89825993593917	1.67830524418160
O	2.83535731042367	-0.78723799384911	0.80204706640407
O	2.29860942288157	-1.02552001356611	2.98164310564599
C	3.70058088955918	-1.02986475989061	3.30363431322856
H	-3.55234993559987	-1.72691243038831	2.94446408625741
H	-5.27358118477533	-1.27897395873332	3.12133542682710
H	-4.78417081509527	-2.47788970982884	1.90850215108751
N	-1.08430527987953	-1.16459145321657	3.39678914704767
H	4.20096441774213	-1.86433880694061	2.81570569467571
H	3.74807516167844	-1.13906971198443	4.38242129347943
H	4.16454870353069	-0.09453491034044	2.99629843136043
H	-2.05042184462066	-0.72895630353187	0.39166719115694
H	1.81367141448839	-0.64979347839553	-0.93237504161343
H	-3.43592312287696	-0.50573976253972	-2.09416288256766
H	1.27489500839310	-0.33587139207243	-3.80912834995823
H	-0.65943159015276	-0.14906961229024	-5.36340246707576
H	-2.96849680419753	-0.23015074257481	-4.52199785915189
H	-3.86046911670602	0.70469518192467	1.94545329479510
C	-1.29236399826582	2.37590046172219	2.15917061598940
S	-2.44836587017340	2.14358703873528	3.55161827397249
H	-1.38775896101157	3.41382579955334	1.84863198496250
H	-1.55254390694299	1.72719068175253	1.32707363427896
H	-0.27070593947856	2.18652857377088	2.47636026653409
H	-2.09239850431744	0.88150190726419	3.84902322914697

Table S1. Energetic properties for stationary points

Stationary point	Total energy (au)	ZPE (au)	ΔE+ZPE 1+2 limit (kcal/mol)
**B3LYP-D3BJ/def2-TZVPP (gas-phase)**			
**1**	**-342.91398464**	**0.13308049**	
**2**	**-1273.61777561**	**0.15182914**	**0.0**
**TS1**	**-1616.51512012**	**0.28962893**	**13.4**
**MIN**	**-1616.56348517**	**0.28599990**	**-19.2**
**INT1**	**-1616.54183752**	**0.28346993**	**-7.2**
**P1**	**-1616.55747978**	**0.28579883**	**-15.6**
**INT2**	**-1616.54992926**	**0.28509232**	**-11.3**
**P2**	**-1616.59032968**	**0.28334201**	**-37.7**
**B3LYP-D3BJ/def2-TZVPP (ethanol)**			
**1**	**-342.92912014**	**0.13374284**	
**2**	**-1273.63490262**	**0.15141048**	**0.0**
**TS1**	**-1616.55291507**	**0.28947631**	**9.7**
**MIN**	**-1616.58597534**	**0.28434668**	**-14.3**
**INT1**	**-1616.57822469**	**0.28425716**	**-9.5**
**P1**	**-1616.58158200**	**0.28475195**	**-11.3**
**INT2**	**-1616.58023126**	**0.28431689**	**-10.7**
**P2**	**-1616.60949778**	**0.28202071**	**-30.5**
**B3LYP-D3BJ/VTZ (gas-phase)**			
**1**	**-342.90143516**	**0.13329061**	
**2**	**-1273.60725807**	**0.15174272**	**0.0**
**TS1**	**-1616.49456383**	**0.28949904**	**11.7**
**MIN**	**-1616.54323167**	**0.28600870**	**-21.0**
**INT1**	**-1616.53218893**	**0.28520927**	**-14.6**
**P1**	**-1616.53696247**	**0.28572328**	**-17.3**
**INT2**	**-1616.52888418**	**0.28489347**	**-12.7**
**P2**	**-1616.57077561**	**0.28321036**	**-40.1**

References

[1] BALIZA, Larissa RSP *et al.* Synthesis and cytotoxic evaluation of heterocyclic compounds by vinylic substitution of ketene dithioacetals. **Chemical Biology & Drug Design**, v. 104, n. 1, p. e14581, 2024.

[2] MISRA, Apoorva et al. A facile one pot synthesis of novel pyrimidine derivatives of 1, 5-benzodiazepines via domino reaction and their antibacterial evaluation. **Journal of microbiological methods**, v. 163, p. 105648, 2019.
